# Persistent inhibition of pore-based cell migration by sub-toxic doses of miuraenamide, an actin filament stabilizer

**DOI:** 10.1038/s41598-017-16759-7

**Published:** 2017-11-27

**Authors:** Christina Moser, Daniel Rüdiger, Florian Förster, Julia von Blume, Peng Yu, Bernhard Kuster, Uli Kazmaier, Angelika M. Vollmar, Stefan Zahler

**Affiliations:** 1 Department of Pharmacy, Ludwig-Maximilians-Universität, Munich, Germany; 2 Max Planck Institute of Biochemistry, Martinsried, Germany; 3 Chair of Proteomics and Bioanalytics, Technical University of Munich, Munich, Germany; 4 Chair of Organic Chemistry, Saarland University, Saarbrücken, Germany

## Abstract

Opposed to tubulin-binding agents, actin-binding small molecules have not yet become part of clinical tumor treatment, most likely due to the fear of general cytotoxicity. Addressing this problem, we investigated the long-term efficacy of sub-toxic doses of miuraenamide, an actin filament stabilizing natural compound, on tumor cell (SKOV3) migration. No cytotoxic effects or persistent morphological changes occurred at a concentration of miuraenamide of 20 nM. After 72 h treatment with this concentration, nuclear stiffness was increased, causing reduced migration through pores in a Boyden chamber, while cell migration and chemotaxis per se were unaltered. A concomitant time-resolved proteomic approach showed down regulation of a protein cluster after 56 h treatment. This cluster correlated best with the Wnt signaling pathway. A further analysis of the actin associated MRTF/SRF signaling showed a surprising reduction of SRF-regulated proteins. In contrast to acute effects of actin-binding compounds on actin at high concentrations, long-term low-dose treatment elicits much more subtle but still functionally relevant changes beyond simple destruction of the cytoskeleton. These range from biophysical parameters to regulation of protein expression, and may help to better understand the complex biology of actin, as well as to initiate alternative regimes for the testing of actin-targeting drugs.

## Introduction

Actin, the most abundant protein in eukaryotic cells, has been mostly associated with migration processes and cell division since its discovery in the 1940´s^1^. This made actin a putative anti-cancer target, and with the advent of actin binding compounds (cytochalasin D 1971^2^, phalloidin 1975^3^, latrunculin 1983^4^, jasplakinolide 1994^5^) the hope for a therapeutic option increased. Since then numerous *in vitro* studies have been conducted with different actin binding compounds, which have greatly improved our understanding of the biology of actin. To date, however, this has not led to a clinically used therapeutic^6^. One might argue that this is due the central role the actin cytoskeleton plays in many cellular processes, and the inevitable and unspecific side effects such an approach might cause. However, the same arguments were raised against the use of tubulin targeting drugs, which have turned out to be a story of success during the last 50 years not only in the treatment of cancer, but also of inflammatory diseases^7^. There are two possible explanations for the lack of advanced preclinical development of actin binding compounds: Firstly, the compounds initially used might indeed have such unfavorable pharmacological profiles that safe *in vivo* application is precluded. Secondly, the approach of using concentrations of compounds eliciting acute cytotoxicity might have been misleading. Concerning the first point, numerous promising compounds have been identified recently^8–10^. Concerning the second point we have learned in the past years that the complexity of actin biology lies much beyond the regulation of overall polymerization and depolymerization^11^.

Consequently, in the present work we have used miuraenamide, an actin filament stabilizing natural compound^9,12,13^ at sub-toxic concentrations and investigated its long-term effects on migration and protein expression patterns of SKOV3 cells.

## Results

### Miuraenamide A (Miu) does not reduce cell viability or proliferation, is subtoxic and does not change the architecture of the actin cytoskeleton at 20 nM

**Figure 1:**
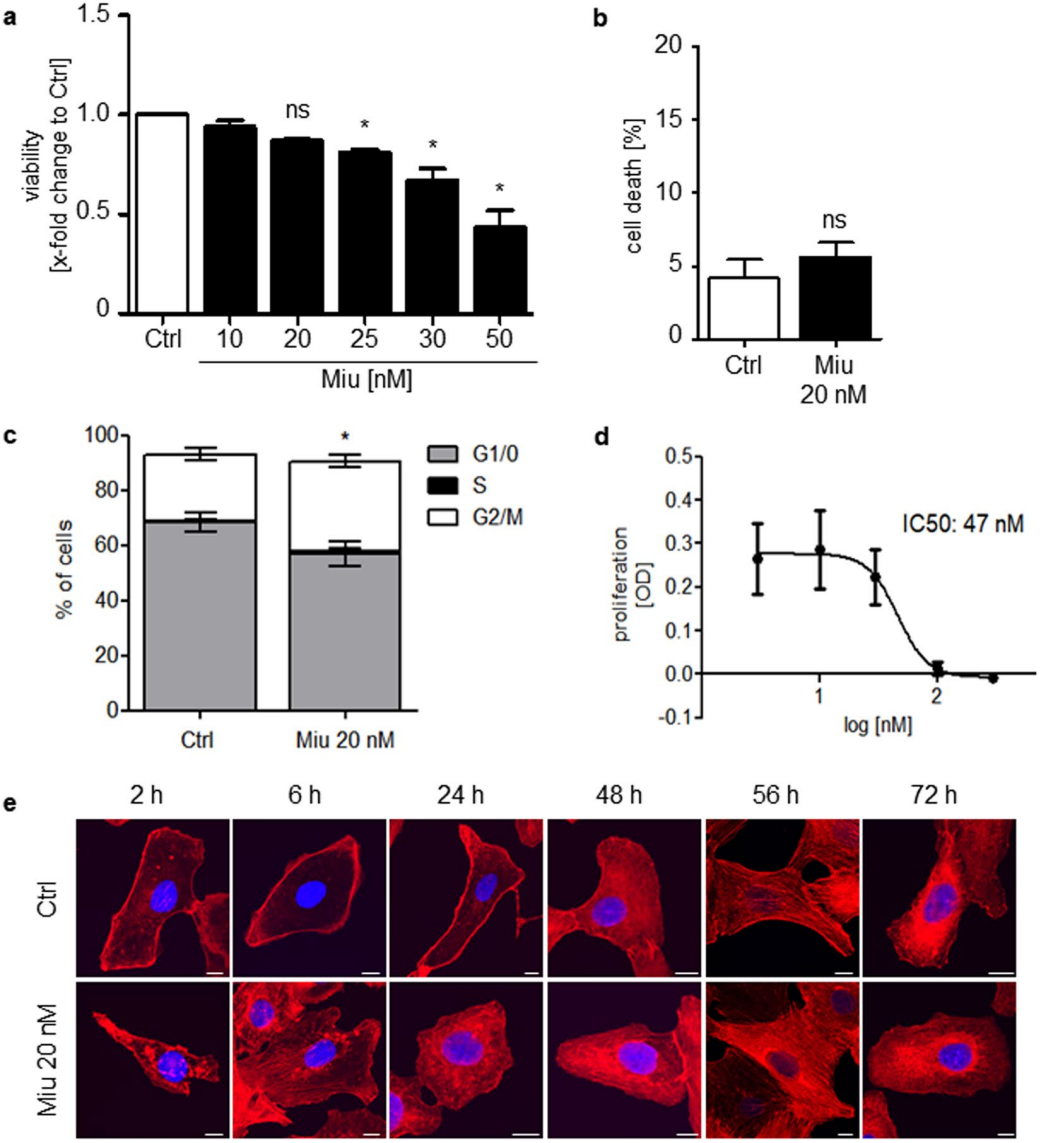
Low dose treatment of SKOV3 with miuraenamide A (Miu) showed no effects on cell viability, proliferation and actin cytoskeleton morphology. (**a**) Cell viability after treatment for 72 h of SKOV3. (**b**) PI exclusion assay after treatment for 72 h with 20 nM Miu. (**c**) Cell cycle analysis. (**d**) Proliferation after treatment with increasing concentrations of Miu. (**e**) Actin staining of SKOV3 cells treated with 20 nM Miu for the indicated time points (rhodamine-phalloidin, red, and nucleus, Hoechst 33342, blue). White bars: 10 µm. ns, not significant. One-Way ANOVA/Bonferroni’s Multiple Comparison Test, *p < 0.05. n = 3.

SKOV3 cells were treated with increasing concentrations of Miu in order to identify a subtoxic concentration. Significant reduction of cell viability was observed starting at concentrations of 25 nM or higher (Fig. 1a). Therefore, the concentration of 20 nM Miu, which showed no induction of apoptosis or cell viability alterations (Fig. 1b), was chosen for analyzing low dose effects of Miu. Analysis of the cell cycle after treatment with 20 nM Miu for 76 h showed only a slight shift to the G2/M phase (Fig. 1c). A dose response curve of Miu treatment in a proliferation assay elicited an IC_50_ value of 47 nM and no significant inhibition at 20 nM (Fig. 1d). The short term treatment with Miu displayed slight agglomerates of actin cytoskeleton in the cytoplasm after 2 h and 6 h. However, over longer periods of treatment (24 h to 72 h) the structure of actin cytoskeleton completely recovered (Fig. 1e).

Human umbilical vein endothelial cells (HUVECs) were used as primary human non-tumor cells in comparison to SKOV3 cells. Only at 50 nM a reduction of viability was observed (Supplementary Figure 3a).

### SKOV3 cell migration through pores is impaired after low dose Miu treatment without affecting other features of migration

**Figure 2:**
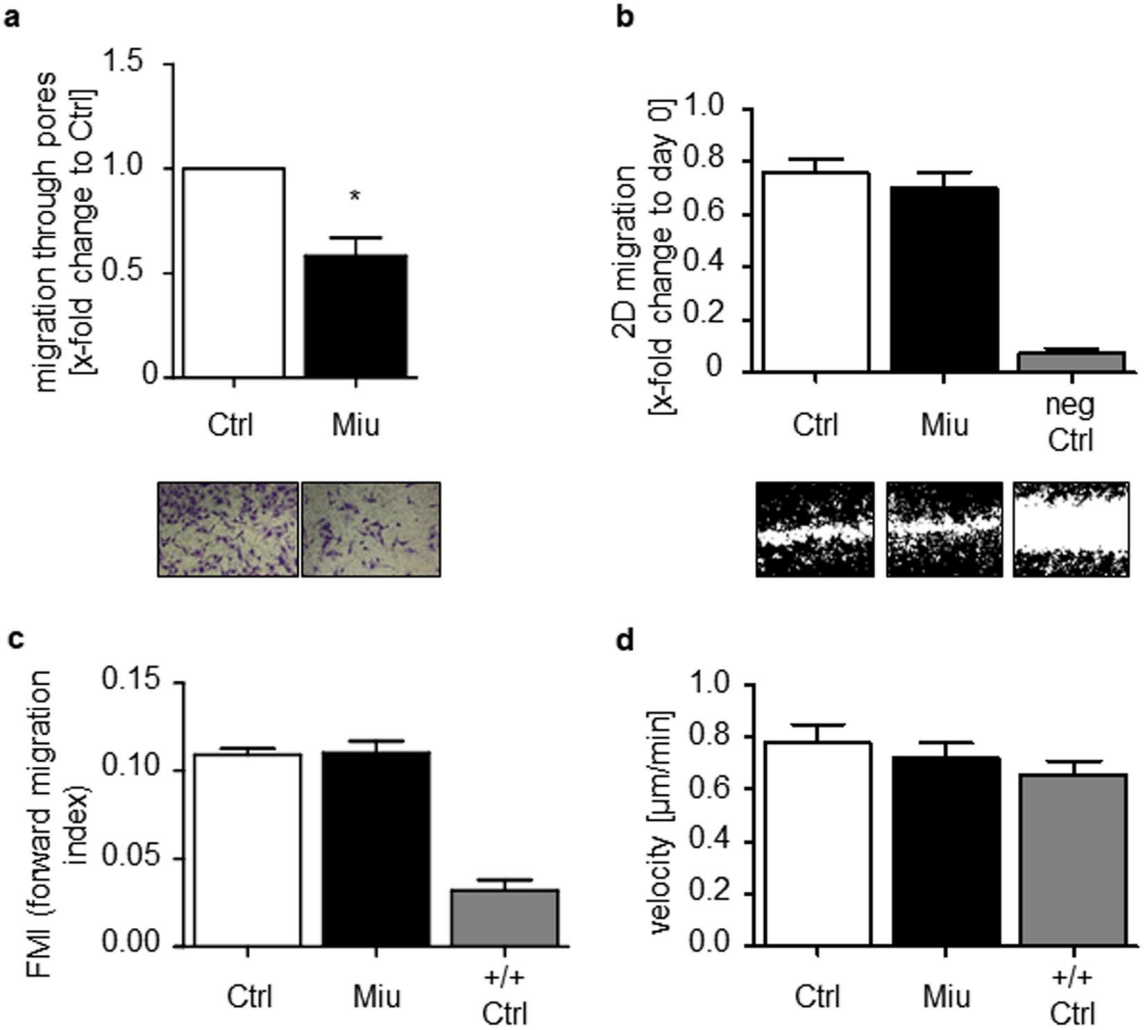
Analysis of SKOV3 cell migration. SKOV3 cells were pre-treated with 20 nM Miu over 72 h, during the migration process no compound was present. (**a**) Boyden chamber migration assay over 24 h migration through a 8 µm pore membrane. Paired two tailed t-test, *p < 0.05. (**b**) Wound healing assay, neg Ctrl: negative control, migration in FCS free medium. One representative image per treatment is shown. (**c**) Chemotaxis assay over 24 h along an FCS gradient. FMI, forward migration index, +/+ Ctrl: positive control, medium with FCS on both sides, no gradient. One-Way ANOVA/Bonferroni’s Multiple Comparison Test, *p < 0.05. n = 3.

**Figure 3:**
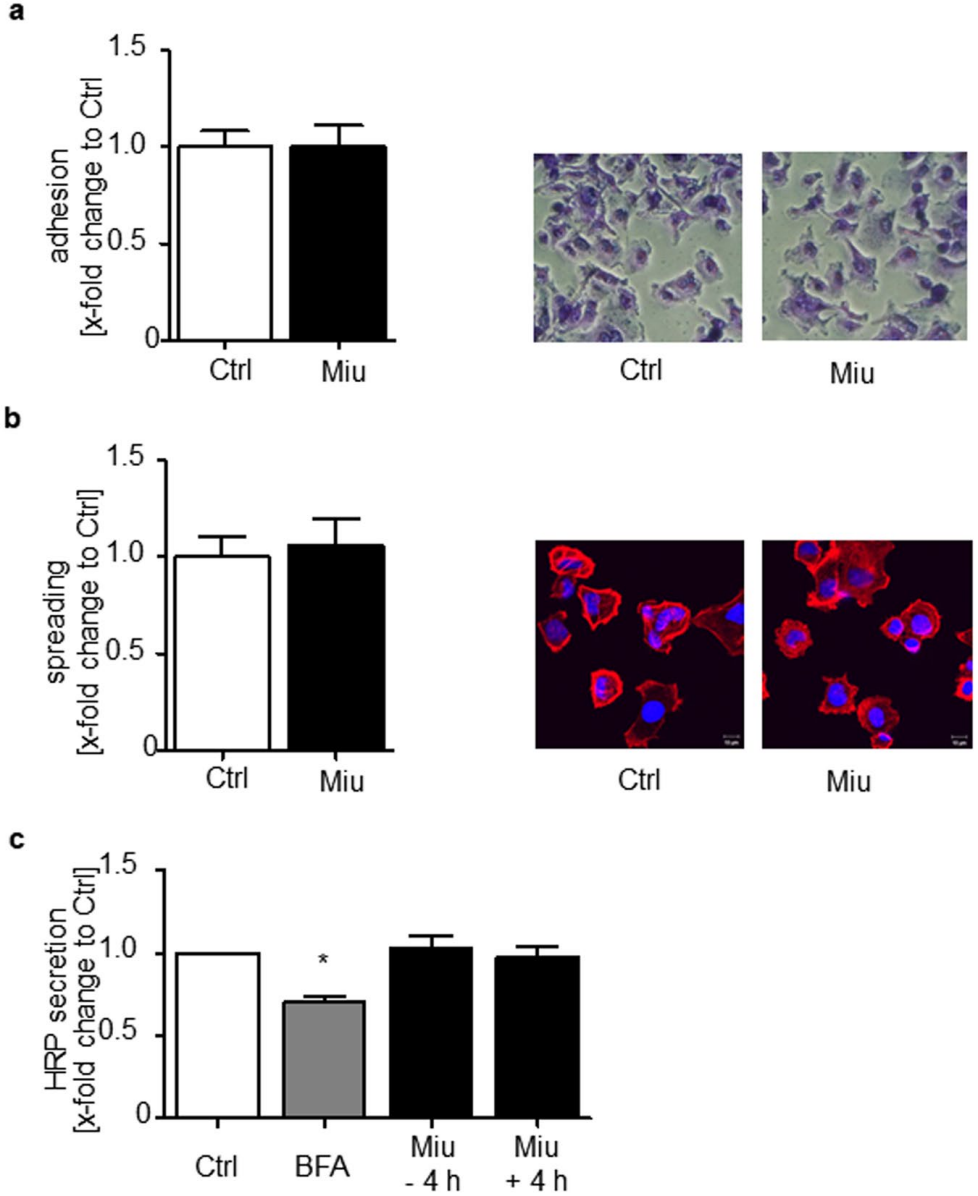
Analysis of migration associated parameters. (**a**) Adhesion and (**b**) Spreading of SKOV3 cells pre-treated with 20 nM Miu over 72 h were quantified after 90 min by counting adhered or spread cells. (**c**) HRP secretion assay with HeLa-ssHRP. Brefeldin A (BFA) was used as positive control, fresh Miu was added ( + 4 h) or not (−4 h) during secretion, n = 4. One-Way ANOVA/Bonferroni’s Multiple Comparison Test, *p < 0.05.

Migration of SKOV3 cells was analyzed in a Boyden chamber assay. Pretreatment with Miu reduced the number of migrating cells nearly by half (Fig. 2a). In contrast, in the wound healing assay, which monitors undirected cell motility as such, migration was not affected by treatment (Fig. 2b). To elucidate the effect of Miu treatment on chemotactic sensing a two dimensional (2D) chemotaxis assay was performed (Fig. 2c). The forward migration index (FMI) of SKOV3 cells and the velocity were not influenced after Miu treatment and thus, showed no significant inhibition of directed migration. Therefore, the inhibitor effect on pore based migration seemed not to be based on an inhibition on motility as such or on a reduced sense of direction. Additional migration associated parameters, like the adhesion and spreading of cells on a surface, which are the initial steps in cell migration, were also unaffected by Miu treatment, both quantitatively and morphologically (Fig. 3a and b). Since intact vesicle trafficking is also mandatory for directional cell movement, we monitored overall cellular secretion in a cell line (HeLa) stably expressing a secreted version of horseradish peroxidase. This was likewise not reduced by treatment with Miu, while the positive control (Brefeldin A) worked as was to be expected. Since both, the 2D chemotaxis and the Boyden chamber assay are based on directional movement, the only difference, which might explain the different outcome in both, is the fact that the cells have to deform and squeeze through small pores in the Boyden chamber.

Interestingly, pretreatment with 20 nM Miu for 72 h did not change migration behavior of HUVECs – neither in the scratch assay, nor in the Boyden chamber (Supplementary Figure 3b and c).

### Nuclear stiffness of SKOV3 cells is enhanced after Miu treatment

**Figure 4:**
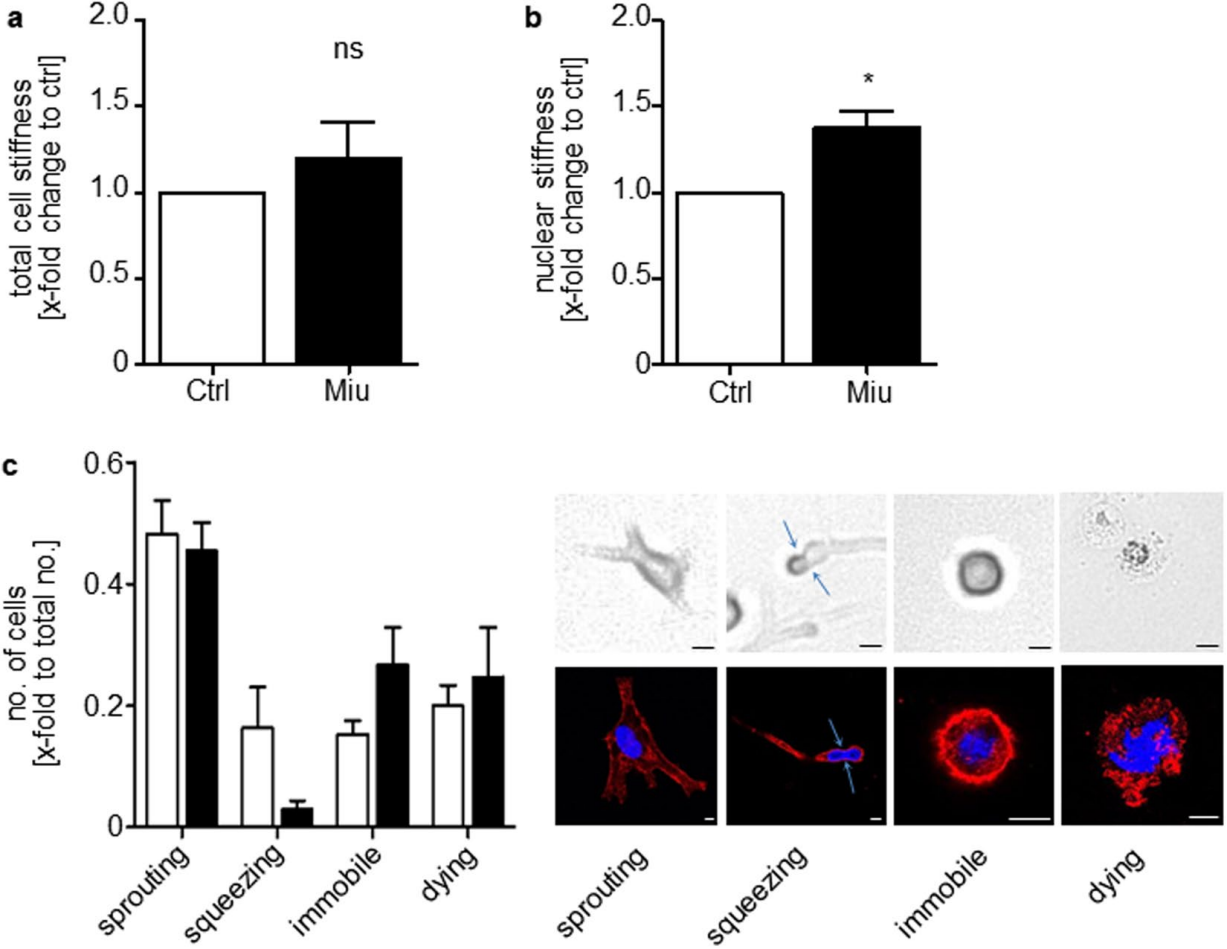
Atomic force measurements of Miu treated SKOV3 cells and Rat tail collagen I 3D gel migration of SKOV3 cells. Cells were treated for 72 h with 20 nM Miu. (**a**) Total cell stiffness was measured with a force map of 80 × 80 µm. (**b**) Nuclear stiffness measurement with single force curves in contact mode, 10–20 cells per experiment were measured. (**c**) Cells were pre-treated for 72 h with 20 nM miuraenamide (Miu) and migrated over 20 h in rat tail collagen I gels (2 mg/ml). Left panel: Quantification of cellular phenotypes normalized to total counted cell number (total no.). Right panel: Example images of cellular phenotypes recorded by life cell imaging with transmitted light microscopy (upper row) or fluorescence microscopy (rhodamine-phalloidin, red, and nucleus, Hoechst 33342, blue, lower row). Bars indicate 10 µm. Arrows indicate the nucleus deformed due to restriction. Paired two tailed t-test, *p < 0.05. ns: not significant, n = 4.

Consequently, in a next step, we probed cell and nuclear stiffness after treatment with Miu by atomic force microscopy as a measure of cell deformability. The integrated cell stiffness was only marginally increased after Miu treatment for 72 h (Fig. 4a). The nucleus is the largest organelle in the cell and is supposed to be the limiting factor during migration through restricted pores, therefore the stiffness of the nucleus was determined separately. The Miu treated cells displayed a 1.4-fold higher stiffness of nuclei compared to control cells (Fig. 4b). In line with higher nuclear stiffness, the three dimensional (3D) migration of SKOV3 cells through a rat tail collagen I gel with restricting mesh size was reduced: fewer cells were able to squeeze through the restrictions on their migration track after Miu treatment. The number of cells which are immobile or die during the observation time slightly increased after Miu treatment by trend, but showed no alteration of sprouting cells, which are able to build protrusions, in comparison to control cells (Fig. 4c).

### Miu treatment causes late downregulation of proteins enriched in the Wnt pathway

**Figure 5:**
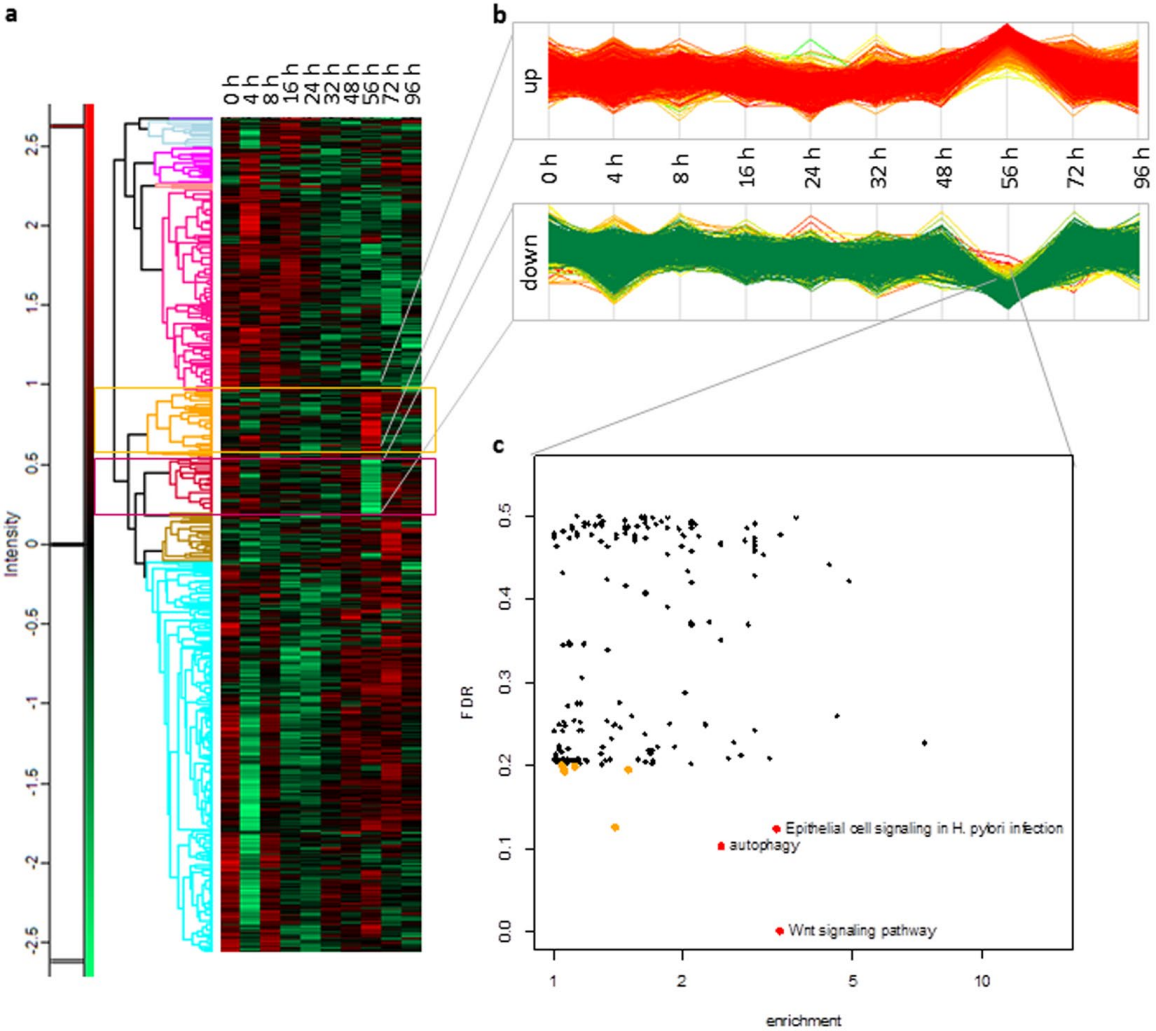
Time course of proteome changes after treatment with Miu. SKOV3 cells were treated with Miu 20 nM for indicated time points. (**a**) Heat plot with ten defined row clusters, red areas indicate up- and green areas indicate down-regulation, log2 scale. (**b**) Defined cluster up- and down-regulated proteins after 56 h treatment depicted in lanes as time series, log2 scale. (**c**) Dot plot of enriched pathways with a threshold of FDR < 0.5 showed down-regulated proteins after 56 h treatment, orange dots: FDR < 0.2, red dots: FDR < 0.2 and enrichment value > 2.

Since actin is an integral part of transcription- and chromatin-remodeling complexes and plays an important role in mechano-induced signaling via the transcriptional co-activators MRTF and YAP, we investigated time dependent changes of the proteome upon treatment with 20 nM Miu. The detected proteins are depicted in a heat plot (Fig. 5a), where clusters downregulated in comparison to control are coded in green and upregulated proteins in red. There are two prominent time windows where larger clusters of proteins are transiently up- or downregulated: after 4 h of stimulation and after 56 h (Fig. 5b). We deemed it much more likely that the latter time point is functionally more important for the effects on nuclear stiffness and migration after 72 h, and, therefore, focused on this cluster for further analysis (Fig. 5b). The regulated proteins in the cluster indicated by a yellow frame in the heat map (upregulated after 56 h, Fig. 5b upper panel), are listed in Supplement Table 1. The proteins of the cluster framed in red (downregulated after 56 h, Fig. 5b lower panel) are listed in Supplement Table 2. These show different regulated pathways in enrichment analysis (Fig. 5c). The KEGG (Kyoto Encyclopedia of Genes and Genomes) Wnt-signaling pathway had a relatively high enrichment value and the lowest false discovery rate (FDR). 17 proteins annotated to this signaling pathway are significantly downregulated in SKOV3 cells after Miu treatment (Supplement Table 3). The next best two pathways were “autophagy” and “epithelial cell signaling in H. pylori infection”. The pathway analysis of the upregulated genes revealed 11 clusters by our criteria (false detection rate <0.2, enrichment >2, Supplementary Figure 2). However, the cluster with the highest enrichment (“sleep”) consisted only of one protein and is functionally not relevant. When we ordered the clusters according to their P-values, “structural constituent of cytoskeleton”and “muscle contraction” were the most significant ones.

### Miu treatment results in the inhibition of MRTF-associated protein expression

**Figure 6:**
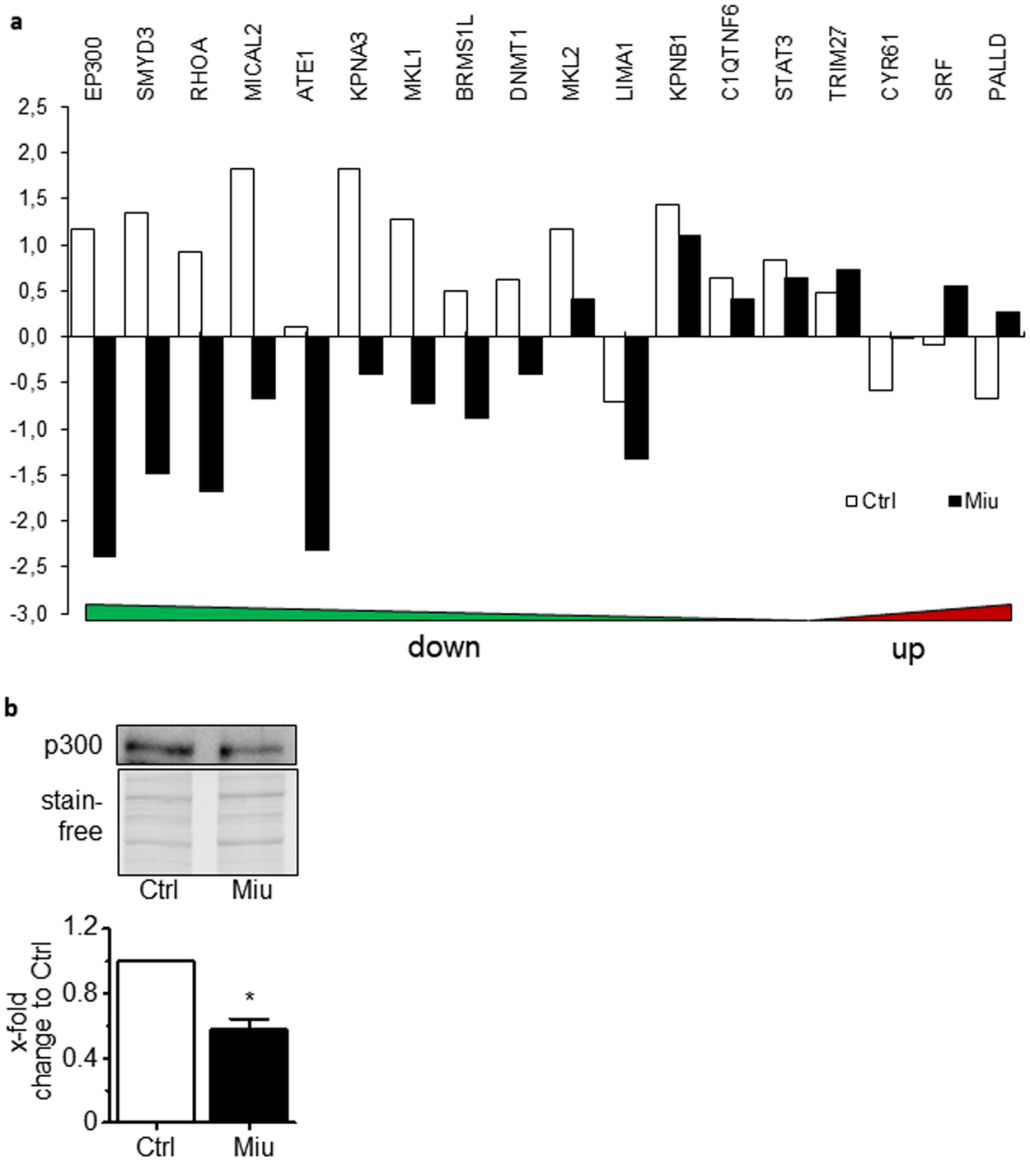
Proteome analysis of MRTF-associated genes and expression of p300 transcription co-activator. SKOV3 cells were treated with 20 nM miuraenamide (Miu) for 56 h. (**a**) MRTF-associated genes were selected by GeneCards® Keyword Search “MRTF”. Miu treated cells (black bars) or harvested at stimulation start point without treatment (Ctrl, white bars) were depicted, log2 scale. b: Protein level of p300 in the Western blot (upper panel with stain free gel as loading control) and quantification (lower panel). One representative image per treatment is shown, n = 4. Paired two tailed t-test, *p < 0.05. The Western blot is cropped; the complete blot is displayed in the Supplementary materials.

Further analysis of the proteome data after 56 h stimulation with Miu was performed concerning MRTF-associated proteins. The selection of genes/proteins was collected by GeneCards^®^ Keyword Search ‘MRTF’ (Supplement Table 4). Most of the listed MRTF-associated proteins were down-regulated after 56 h (Fig. 6a). A few of them are related to migration regulating processes and interact with MRTF signaling. The acetyltransferase and transcription co-activator p300 was strongly down-regulated in proteome analysis after 56 h treatment with Miu. This was exemplarily confirmed by Western blot analysis, which indicated a reduction of protein level to 0.58-fold compared to control (Fig. 6b).

## Discussion

Though intuitively attractive as an anti-tumor target, actin has been neglected in this regard for many years due to initial failures^6^ and bad therapeutic indices^14^. Recently, we successfully used chondramide, an actin nucleating myxobacteriual depsipeptide^15^ for inhibiting tumor growth^16,17^ and metastasis^18^ *in vivo*. Furthermore, by using chondramide, we elicited very specific effects on the differentiation of macrophages^19^ or kinase signaling^16^ at concentrations were an obvious destruction of the actin cytoskeleton and acute cytotoxic effects were still absent. This potential selectivity of actin binding compounds can maybe be explained by the concept that actin is not merely forming polymers with other actins and subsequently depolymerizes again, but that there is a continuous competition between actin binding proteins for binding sites on actin^11^. This complex network then allows for subtle control of the “actin-interactome” and related cell functions. With respect to actin binding small molecular compounds it has been found that they, in turn, can compete with specific actin binding proteins. So, for example, kabiramide C has been shown to compete with actin capping proteins like gelsolin^20^.

These findings prompted us to investigate the actions of miuraenamide A, an actin filament stabilizing myxobacterial compound^12^, which is synthetically relatively well accessible^9^, on tumor cell migration at sub-toxic concentrations. We focused on two issues: 1) biomechanical changes, and 2) changes in the proteome of treated cells over time.

Concerning the first aspect, we found that at a concentration of 20 nM, which did not cause changes in cell viability, the structure of actin was initially subtly changed, but recovered subsequently. At this later time (72 h) migration of cells and chemotaxis were unaffected in 2D-settings, while the migration through pores (or a collagen mesh with restricting size) posing a spatial obstacle was reduced. This was accompanied by a small rise of overall mechanical cell stiffness, but a significant increase of nuclear stiffness. This is in line with recent findings, which identified the nucleus as the most relevant cellular barrier while navigating through confined environments^21^. While the stiffness of the nuclear envelope itself is mainly determined by the lamins^22^, actin also plays an important role here: on the one hand, it is needed to deform the nucleus^23^, on the other hand, deformation bears the danger of nuclear rupture^24^, and actin is needed to form a mechanical shield for the nucleus^25^. These different actions are, again, regulated by different actin binding proteins: Arp2/3^23^ and FMN2^25^, respectively. It lies close at hand that miuraenamide could change nuclear deformability by entering the competitive process for actin binding. In addition, it has recently been shown that the recruitment of different actin pools by different nucleating proteins (Arp2/3 vs. mDia) causes distinct migration behavior in cells^26^. Processes like these might explain, why we only see effects in a specific migration mode. This aspect is supported by the finding that migration in confined and low-adhesive environment (which is clearly the case in migration through pores of a Boyden chamber) elicits a different migration mode^27^. Interestingly, we did not observe this reduction of pore based migration in non-tumor cells (HUVECs). At the moment we can only speculate about the mechanistic reason for this difference between cell types. Different kinetics of actin turnover, or different relative importance of signaling pathways due to cell type could be responsible.

Concerning the second aspect, changes in the proteome due to long term treatment, there is a close relation to nuclear stiffness or deformation: changes of this parameter change gene expression^28^. Furthermore, mechanosensitive transcriptional co-activators like MRTF or YAP are closely regulated by changes in the actin equilibrium^29,30^. Since this is also a process of competition for actin binding sites^30^, we looked for effects of miuraenamide treatment on protein levels. We focused on changes which occurred in the time window briefly before the migration experiment, since we deemed these to be the functionally most relevant ones. After 56 h two very distinctly up- or downregulated protein clusters emerged. Looking for causes for the reduced migration we first concentrated on the down-regulated proteins (Supplement Table 1) and analyzed for pathways with significantly enriched regulated proteins according to KEGG (Kyoto Encyclopedia of Genes and Genomes) pathways. The three signaling pathways with the combination highest enrichment /lowest false discovery rate were: 1) “Epithelial cell signaling in H. pylori infection”, which is not specifically related to migration, but contains a number of motility associated genes, 2) “Autophagy”, which is interesting, since actin has recently been shown to be related to autophagy^31^, and since autophagy influences migration^32^, and 3) “Wnt-signaling pathway”, which is closely related to migration. The protein with the highest downregulation in the Wnt pathway turned out to be the histone acetyl transferase EP300 (or p300), which has already been linked to the mechanosensitive SRF/MRTF pathway^33^. This is surprising, since actin polymerization should cause an increased nuclear translocation of MRTF^34–36^, and, consequently, upregulation of MRTF target genes. However, when looking for MRTF target gene products, we surprisingly found some classical candidates downregulated after treatment with miuraenamide at 56 h (Supplement Table 4). Like with the recovery of the microscopic architecture of the actin cytoskeleton after some hours of low-dose miuraenamide, also MRTF signaling seems to be counter-regulated after some time by an as yet unknown mechanism. A closer look at the upregulated proteins indicated “structural constituent of cytoskeleton” and “muscle contraction” as the most significantly influenced pathways. This could be interpreted as a compensatory reaction of the cells upon the manipulation of actin by Miu.

All in all, the proteomics data do not explain the changes in nuclear stiffness by treatment with miuraenamide, since no proteins connected to the nuclear lamina are altered. The mode of action might depend on the direct effects on actin and/or actin binding proteins. The changes in protein expression seem more like a consequence of the nuclear effects than a cause for them and might indicate positive or negative feedback mechanisms.

In conclusion, low-dose treatment of tumor cells with miuraenamide over prolonged time does not cause cytotoxic effects, but inhibits migration through confined environments in a multifactorial way: nuclear stiffness is increased and, at the same time, the proteome is altered in several anti-migratory ways (especially decreased Wnt- and MRTF-signaling). It is tempting to speculate that by using the right treatment scheme it could be possible to apply actin binding compounds for eliciting specific and subtle changes in cellular behavior. It seems worthwhile to revisit some of the actin compounds yet neglected for *in vivo* studies under this aspect.

## Methods

### Study compound

Miuraenamide A was synthetized as described previously^9^.

### Cultivation of cell lines

The human ovarian cell line SKOV3 was purchased from LGC Standards (ATCC-HTB-77, Wesel, Germany) and was cultivated in RPMI 1640 medium (PAN Biotech, Aidenbach, Germany) with 1% penicillin/streptomycin (1.5 mM, PAA Laboratories, Austria) and 10% (v/v) FCS (PAA Laboratories, Pasching, Austria). Absence of mycoplasms was regularly tested by PCR. Cells were used in passages 5–10 after thawing. Creation of the stably transfected HeLa-ssHRP cells was described previously^37^. These cells were cultivated in DMEM high glucose (PAN Biotech, Aidenbach, Germany) with 1% penicillin/streptomycin (1.5 mM) and 10% FCS. The cells were cultivated in an incubator with constant humidity at 37 °C and 5% CO_2_. Primary human umbilical vein endothelial cells (HUVECs) were purchased from Promocell (Heidelberg, Germany) and cultivated in endothelial cell growth medium (ECGM) from Promocell, according to the manufacturer´s instructions. Cells were routinely used in passage 4 to 6.

For all experiments samples were collected simultaneously after staggered treatment.

### Proliferation assay

The proliferation assay was performed in 96-well plates with an initial concentration of 5 × 10^3^ cells/well. Cells were seeded overnight and afterwards treated for 72 h with the indicated concentrations of compound. Additional wells seeded with untreated cells were measured as day zero control. The cells were fixed and stained, after a washing step with PBS, with crystal violet/methanol (0.5% crystal violet (w/v), 20% methanol (v/v)). After elution of crystal violet with ethanol/Na-citrate (50% ethanol (v/v), 50% 0.1 M Na-citrat (w/v)), the absorption of the solution was determined using a microplate reader at 540 nm (Tecan Sunrise^TM^ Microplate Absorbance Reader, Maennedorf, Austria).

### CellTiter-Blue (CTB) viability assay

To analyze the viability of cells, they were seeded in 96-well plates o/n. The cells were treated with the indicated compounds for 72 h or measured for day zero control. The viability of cells was determined by incubation with CellTiter-Blue® (CTB, Promega, Mannheim, Germany) reagent for 2 h. Fluorescence was measured with a microplate reader at 550_Ex_/595_Em_ (Tecan SpectraFluor plus^TM^ Microplate Reader, Maennedorf, Austria).

### Propidium iodide (PI) exclusion assay

The cancer cells were cultivated in twelve-well plates with a concentration of 4 × 10^4^ cells/well, stimulated with miuraenamide A, and subsequently harvested by trypsination. After a washing step with PBS, the cells were exposed to a solution of propidium iodide (PI, 5 µg/ml) in PBS. Subsequently, cells were analyzed immediately by flow cytometry using FACSCanto^TM^ II (BD Biosiences, Heidelberg, Germany).

### Cell cycle analysis

Cells were incubated with 20 nM miuraenamide for 72 h. Subsequently, cells were harvested on ice and incubated in a hypotonic buffer (0.1% sodium citrate, 0.1% Triton X-100 and 50 µg/ml propidium iodide) overnight at 4 °C, and then analyzed by flow cytometry on a FACSCalibur (Becton Dickinson, Heidelberg, Germany) using Cell Quest Pro Software (Becton Dickinson, Heidelberg, Germany). The percentage of living cells in G0/G1, S and G2/M phase was evaluated after gating out cell debris, using the Watson pragmatic model for cell cycle analysis in the FlowJo software (Tree Star Inc., Ashland, OR, USA).

### Wound healing assay

The wound healing or scratch assay was performed with confluent cell layers. Cells were pre-treated for 72 h with the indicated compound concentrations, seeded into 96-well plates and incubated for minimum five hours at 37 °C. Scratch was executed with a pipette tip and after 14 h of migration, cells were stained with crystal violet/methanol. Pictures of the scratch were taken with an inverted microscope (Eclipse Ti, Nikon, Duesseldorf, Germany). The cell-covered area was quantified by ImageJ 1.51 f and normalized to day 0.

### Boyden chamber assay

Migration through restricted pores towards an FCS gradient was determined using the Boyden chamber migration assay. Briefly, the cells were pre-stimulated for 72 h with the indicated compound concentration in culture dishes. Subsequently, cells were detached and 2 × 10^4^ cells were seeded without compound in FCS negative medium in the upper compartment of the chamber. The lower compartment was filled with medium containing 10% FCS to generate a chemotactic gradient. After migration for 20 h at 37 °C and 5% CO_2_, cells were fixed and stained with crystal violet/methanol. Remaining cells in the upper compartment were removed with q tips and pictures of the bottom side of the membrane were taken with an Axiovert 25 microscope (Zeiss, Jena, Germany) and a Canon EOS 450 C camera (Canon, Krefeld, Germany). The amount of migrated cells was analyzed with ImageJ.

### Adhesion assay

The adhesion assay was performed with pre-treated cells in 96-well plates. A concentration of 4 × 10^4^ cells/well were seeded on collagen G coated wells and allowed to adhere for 90 min. Cells were stained with crystal violet/methanol and imaged with Axiovert 25 microscope and adhering cells were counted.

### Chemotaxis assay

With the chemotaxis assay, migration of cells along an FCS gradient without spatial restriction was detected. Cells were pre-treated with the indicated compound concentration and seeded into ibidi^TM^ µ-Slide Chemotaxis, collagen IV coated (ibidi, Munich, Germany) in compound free medium. After the cells had attached to the chamber slide, the channel was washed with medium without FCS and a gradient was generated by filling one reservoir with FCS negative medium and the other with 10% FCS medium. For negative control 10% FCS was filled in both reservoirs, which generates an environment of undirected migration. Images were taken every ten minutes and the indicated migration parameters were calculated using the chemotaxis and migration tool Qt Open Source Edition version 4.3.2.

### Three dimensional (3D) collagen I gel migration

Rat tail collagen I gels were prepared according to the manufacturer’s protocol (ibidi, Munich, Germany) with: RPMI 1640 10x (10 µl), NaOH 1 M (3 µl), H_2_O (24.5 µl), NaHCO_3_ 7.5% (2.5 µl), RPMI 1640 1x (25 µl), Rat tail collagen I, 5 mg/ml (60 µl), SKOV3 cells 0.25 × 10^6^ (25 µl). Briefly, gels with a concentration of 2 mg/ml were mixed on ice with 0.25 × 10^6^ cell/mixture. Rat tail collagen I was added at the end and immediately pipetted into ibidi^TM^ µ-Slide Chemotaxis. For gelation, the gel was left on ice for 15 min before incubation for 1 h at 37 °C, 5% CO_2_. The chemotaxis reservoirs were filled with medium with or without 10% FCS to create a chemotactic gradient. Life cell images were taken using an inverted microscope (Eclipse Ti). Images were taken with a 10x phase contrast objective every ten minutes over 20 h and analyzed with the chemotaxis plugin in ImageJ.

### HRP secretion assay

HeLa-ssHRP (signal sequence horseradish peroxidase) cells constitutively expressing a secretable form of horseradish peroxidase (HRP)^37^ were seeded and pre-stimulated as described before. After an indicated stimulation period, the medium was removed and the cells were washed five times with fresh medium. Finally, the medium was exchanged against 300 µl of fresh medium with or without compound. The secretion inhibitor Brefeldin A (Sigma Aldrich, Taufkirchen, Germany) was used as a positive control. Secretion was allowed in an incubator over 4 h, before harvesting the cells. The medium was collected as secretion probe, while cells were trypsinated (PAN Biotech, Aidenbach, Germany) and collected by centrifugation. The cell pellets were lysed with 0.5% Triton-X 100 (Merck, Darmstadt, Germany) in PBS on ice for 20 min. After an additional centrifugation at 4 °C, 14000x g for 20 min, supernatants and pellet fraction were transferred to a 96-well plate. The HRP substrate 2,2′-azino-bis((3-ethylbenzthiazoline-6-sulfonic acid), Sigma-Aldrich, Taufkirchen, Germany)) was added to the samples and the absorbance was read at 405 nm using a Tecan Microplate Reader (Tecan, Maennedorf, Austria). The signal of the supernatant was normalized to the signal in the pellet fraction.

### Confocal laser scanning microscopy

The cells were seeded in ibidi^TM^ μ-Slides, cultivated o/n and treated for indicated time points. Afterwards they were permeabilized with Triton X-100 0.1% (v/v), blocked with BSA (Sigma Aldrich, Taufkirchen, Germany) 1% or 5% and stained with rhodamine-phalloidin (1:400, Life Technologies, Darmstadt, Germany) and Hoechst 33342 (5 µg/ml, Sigma Aldrich, Taufkirchen, Germany) diluted in BSA 1% and incubated for 1 h at RT or o/n at 4 °C. After two washing steps with PBS Ca^2+^/Mg^2+^ (pH 7.4) samples were mounted (FluorSave^TM^, Merck, Darmstadt, Germany). Images were taken with a Leica TCS SP8 SMD (Leica Microsystem, Wetzlar, Germany) using a 40x oil immersion lense.

### Atomic force microscopy

Cells were seeded into 34 mm dishes and stimulated for 72 h with the indicated concentrations of compound. The stiffness of the total cell surface and the specific nuclear stiffness were measured with an atomic force microscope (NanoWizard® 4, JPK Instruments, Berlin, Germany). Briefly, cantilevers (MLCT-E: k = 0.1 N/m, f = 38 kHz; MLCT-C: k = 0.01 N/m; f = 7 kHz; silicon nitride; front angle 15 ± 2.5°; quadratic pyramid tip shape) were calibrated with the contact-free method and used for measurements in the contact mode. Total cellular stiffness was determined by a force map with an area of 80 × 80 µm, whereas stiffness of the nucleus was measured by single force curves. For both measurements the velocity was 2 µm/s and the set point was 1 nN. Data were analyzed with the NanoWizard® Data Processing software version 6.0.50 and the stiffness was calculated according the Hertzian contact model (Young’s modulus).

### Western blot analysis

Proteins were separated by SDS-PAGE, transferred to a nitrocellulose membrane and blocked with 5% BSA for 1 h at RT. The membrane was incubated with primary antibody p300 (NM-1), diluted in BSA 5% (1:1000, ThermoFischer Scientific, Germering, Germany), afterwards the membranes were incubated with secondary HRP-conjugated antibody (1:10000, HRP, Goat-Anti-Mouse IgG1, Biozol Diagnostica, Eching, Germany) for 1 h at RT. The chemiluminescence signal was detected with a ChemiDoc™ Touch Imaging System (Bio-Rad Laboratories, Munich, Germany).

### Proteomic sample processing and measurement

Cells were seeded in 10 cm dishes and treated with 20 nM miuraenamide for 4, 8, 16, 24, 32, 48, 56, 72 and 96 h and harvested simultaneously. We can not completely exclude that some cyclic behavior of the cells (e.g. depending on cell cycle) might have altered protein expression in addition to treatment. Subsequently, samples were lysed in 8 M urea with 40 mm Tris/HCl (pH 7.6), 1 × EDTA-free protease inhibitor cocktail (Complete Mini, Roche), and 1 × phosphatase inhibitor mixture (Sigma-Aldrich). The lysate was centrifuged at 10,000 × g for 10 min and the supernatant was kept at −80 °C before further processing. It was then reduced with 10 mM DTT and alkylated with 50 mM chloroacetamide before digested overnight with trypsin (Promega, Madison, WI). The digest was then desalted with C18 SepPak columns (Waters, MA). TMT labeling of desalted digest was carried out with TMT10 reagents (ThermoFisher Scientific) according to the manufacturer’s instruction. Peptide mixture was then fractionated with a Trinity P1 column on a Dionex Ultimate 3000 HPLC system (both ThermoFisher Scientific) into 32 fractions. These fractions were then vacuum dried and reconstituted in 0.1% formic acid (FA) for LC-MS/MS measurement.

The samples were measured on a Dionex Ultimate 3000 nanoLC (ThermoFisher Scientific) coupled to an Orbitrap Q Exactive HF mass spectrometer (ThermoFisher Scientific). NanoLC separation was carried out using an in-house packed capillary column (75 μm × 45 cm) filled with 3 μm Reprosil Gold C18 particles (Dr. Maisch GmbH, Ammerbuch, Germany) at 300 nL·min-1. Sample was loaded onto a trap column (75 μm × 2 cm) in 0.1% formic acid at 5 μL·min−1 for 10 min. The trap column was packed with 5 μm Reprosil ODS-3 particles. The analytical column was heated to 50 °C using a 30-cm capillary column heater (ASI, Pompton Plains, NJ). Solvent A was 0.1% FA, 5% DMSO in water; solvent B was 0.1% FA, 5% DMSO in acetonitrile^38,39^. For the analysis of regular peptides, the gradient was 0–1 min, 2–4% B; 1–52 min, 4–32% B; 52–54 min, 35–80% B; 54–56 min, 80% B; 56–58 min, 80–2% B; 58–60 min, 2% B. A top 25 data dependent acquisition method was used for MS. The survey scan was acquired at 60,000 resolution with a mass range of 360–1300 m/z and AGC target value of 3e6. The maximum injection time (max IT) was 100 ms. MS/MS spectra were acquired at 30,000 resolution with fixed first mass at 120 m/z. AGC target was 2e5 and max IT was 57 ms. Isolation window was 1.7 m/z and normalized collision (NCE) energy was 33. An underfill ratio of 1.0% was used and + 1, + 7 and higher, and unknown charge states were excluded. Further setting included “peptide match preferred” and the “exclude isotopes” option was turned on. Dynamic exclusion was set to 20 s.

The raw files were searched using MaxQuant (v1.5.3.30) against the UniProtKB Human Reference Proteome database (v22.07.13, 88,381 entries). Default MaxQuant parameters were used and the match-between-runs feature was enabled. The results were further processed in Perseus (v1.5.5.3). The detected proteins were grouped into ten clusters based on Pearson correlation. The clusters with down- and up-regulated proteins after stimulation for 56 h were depicted in plots with the statistic software R (R version 3.3.2). GOBPs (Gene Ontology and Biological Pathways) and KEGG (Kyoto Encyclopedia of Genes and Genomes) pathways were plotted with a threshold of FDR < 0.5. Pathway with FDR < 0.2 (plotted in orange) and the highest enrichment values (enrichment > 2) were depicted in red with annotations.

### Statistics

The experiments were performed three times on three different days and done in triplicates each, if not otherwise noted. One-way ANOVA test with relevant posttest (Dunnett’s Multiple Comparisons or Bonferroni’s Multiple Comparison Test) or paired two tailed t-test were used to assess the significance between treatment groups or pairs. The statistical analysis was conducted with GraphPad Prism 5 and Microsoft Excel.

### Data availability

The datasets generated during and/or analysed during the current study are available from the corresponding author on reasonable request.

## Electronic supplementary material

Supplementary data
