# Supplementary material for: Persistent inhibition of pore-based cell migration by sub-toxic doses of miuraenamide, an actin filament stabilizer
Source: Sci Rep. 2017 Nov 27;7:16407. doi: 10.1038/s41598-017-16759-7 (PMC5703899; doi:10.1038/s41598-017-16759-7)

Persistent inhibition of pore-based cell migration by sub-toxic doses of miuraenamide, an actin filament stabilizer

Christina Moser[1], Daniel Rüdiger[1], Florian Förster[1], Julia von Blume[2], Peng Yu[3], Bernhard Küster[3], Uli Kazmaier[4], Angelika M. Vollmar[1] and Stefan Zahler[1]

[1] Department of Pharmacy, Ludwig-Maximilians-Universität, Munich, Germany

[2] Max Planck Institute of Biochemistry, Martinsried, Germany

[3] Chair of Proteomics and Bioanalytics, Technical University of Munich, Munich, Germany

[4] Chair of Organic Chemistry, Saarland University, Saarbrücken, Germany

Supplement Table 1: Red cluster of proteome analysis (downregulated at 56 h, log2 scale).

Gene symbol	Ctrl	56h
AAMDC	-0,193	-2,333
ABCC1	1,086	-1,780
BHD10	0,986	-0,932
ABHD5	1,362	-1,824
ABLIM1	-0,570	-1,542
ABT1	0,569	-1,713
ABTB1	-0,807	-1,652
ACP6	0,347	-2,505
ADD2	0,626	-2,356
ADHFE1	1,332	-1,549
AGO3	0,168	-2,320
AIDA	0,922	-0,862
AIM1	0,353	-1,290
AKAP10	0,248	-2,165
AKR7A2	0,956	-1,231
ALDH2	-0,092	-0,352
ALG1	0,388	-1,339
AMBRA1	1,643	-0,982
ANAPC2	1,149	-2,066
ANAPC7	1,364	-2,336
ANKS1A	1,399	-0,645
ANKS3	0,736	-2,426
AOC1	0,958	-2,085
AP3M2	1,241	-1,988
AP3S1	1,301	-1,912
AP4B1	0,704	-1,396
APIP	0,809	-1,576
APOA1BP	-0,432	-1,541
APP	0,251	-1,553
ARF5	0,858	-1,476
ARHGAP23	0,615	-1,712
ARHGEF7	0,471	-2,350
ARIH2	0,461	-2,099
ARL14EP	1,121	-1,852
ARL6IP5	1,142	-2,394
ASMTL	-0,253	-1,692
ATE1	0,110	-2,319
ATG10	0,877	-1,445
ATG101	1,774	-2,012
ATG12	0,415	-2,428
ATG16L1	0,361	-1,480

Gene symbol	Ctrl	56h
ATP1A1	0,414	-0,244
ATP2C1	-0,200	-2,498
ATP5I	0,147	-2,707
ATP6V1B2	0,391	-1,910
ATP6V1G1	-0,175	-2,365
ATXN2	0,991	-1,356
B3GAT1	0,581	-2,459
BAX	0,874	-2,472
BBS2	0,340	-1,495
BCAS2	0,732	-2,550
BIN3	0,519	-2,203
BLMH	0,850	-2,248
BMP2K	0,165	-1,443
BMS1	1,518	-1,379
BOD1L1	-0,014	-0,799
BOLA3	1,007	-2,021
BRCC3	0,473	-1,891
BUD13	0,877	-2,566
C14orf2;MP68	0,830	-1,952
C17orf49;BAP18;RNASEK-C17orf49	0,383	-0,878
C2orf49	0,950	-1,333
C6orf106	-0,711	-0,877
C9orf41	1,075	-2,068
CABLES1	0,477	-2,432
CAMK1	0,460	-1,887
CASP10	0,805	-1,676
CCDC138	1,957	-1,080
CCDC18	2,239	-1,285
CCDC53	0,602	-2,713
CCNDBP1	0,336	-2,220
CDIPT	0,482	-1,023
CDK11A	-0,148	-1,463
CDYL	1,578	-1,923
CENPT	1,744	-1,360
CEP162	-0,437	-1,395
CES1	-0,228	-1,572
CFLAR	-0,471	-1,617
CHD6	0,664	-2,318
CHD8	0,549	-2,500
CHMP5	0,075	-2,266
CIR1	0,974	-2,223
CLK2	-1,377	-1,817
CMC1	0,738	-2,445
CMPK1	-0,029	-2,412

Gene symbol	Ctrl	56h	Gene symbol	Ctrl	56h
CMTR2	1,755	-1,596	EPB41L1	-1,002	-0,683
CNN3	0,785	-1,600	EPG5	0,067	-1,429
COA6	-1,263	-1,727	EPN1	0,374	-1,474
COMMD6	0,087	-2,588	EPN2	0,736	-0,897
COPS3	1,863	-1,770	ERCC4	1,310	-1,747
CPEB2	-0,289	-2,399	EVL	0,347	-1,883
CPSF6	1,551	-1,569	EXOC8	1,848	-1,848
CRELD2	0,560	-1,957	FAHD2A;FAHD2B	1,116	-0,817
CREM	0,495	-2,144	FAM109A	2,574	-1,171
CRKL	1,017	-2,462	FAM111A	1,860	-2,017
CROT	0,504	-2,205	FAM114A1	-0,217	-1,996
CSNK1A1	1,030	-2,068	FAM168B	0,673	-2,337
CSNK1D	1,141	-1,936	FAM174B	0,585	-1,697
CSNK1E	-1,380	-0,968	FAM193A	0,472	-2,188
CSNK2A2	1,081	-1,736	FAM20B	0,300	-1,896
CSRP2	1,363	-1,909	FAM219A	1,162	-2,320
CTNNBIP1	0,344	-2,751	FAM96B	1,294	-2,439
CTSH	-0,086	-2,422	FANCM	-0,571	-1,703
CUTC	0,537	-2,427	FBXL8	-0,243	-2,166
CYB5R4	0,507	-2,463	FBXO42	0,924	-1,230
DCAF6	0,531	-1,581	FCHSD2	0,967	-1,363
DCTN4	-0,129	-1,121	FKBP2	-0,518	-2,011
DCUN1D5	1,061	-2,448	FLII	1,130	0,402
DDAH1	1,175	-1,448	FLNC	-0,881	-1,821
DDI2	1,421	-2,028	FOXE1	0,674	-1,002
DESI1	1,224	-2,311	FOXO6;FOXO4;FOXO1	0,569	-2,433
DHRS4;DHRS4L2	0,480	-1,493	GALNT7	0,659	-2,595
DHRS7B	0,746	-1,865	GAMT	-0,660	-1,857
DNAJC8	1,480	-1,129	GCC2	0,537	-2,080
DNPEP	0,386	-2,578	GEMIN2	0,870	-1,955
DOCK11	0,263	-2,345	GIT1	1,806	-0,713
DPYD	0,189	-2,403	GLCCI1	-0,336	-1,760
DTX2	0,528	-2,211	GLG1	1,105	-1,947
DUSP12	1,316	-1,989	GLS	1,104	0,675
DVL3	1,324	-1,681	GM2A	0,393	-0,557
E2F7	2,028	-1,963	GNB1L	-0,762	-1,806
EAPP	0,967	-2,338	GNB5	0,929	-2,596
ECI2	0,942	-0,600	GOLGA1	1,913	-1,460
EED	1,606	-1,826	GORASP2	0,430	-2,430
EFHC1	0,749	-1,840	GOSR1	0,168	-2,055
EHD3	0,327	-2,480	GPCPD1	1,123	-2,319
EIF3I	1,250	-1,716	GPHN	0,357	-1,906
EIF4E2	0,668	-2,519	GPN3	0,034	-2,249
ELMO1	0,507	-2,349	GSN	-1,456	0,107
EP300	1,169	-2,379	GSTK1	0,282	-1,427

Gene symbol	Ctrl	56h
GSTZ1	0,446	-2,046
GTDC1	0,998	-1,463
HARS	-0,610	-0,463
HCCS	1,260	-1,099
HDAC2	0,960	-1,726
HDHD1	1,535	-0,940
HIPK3	1,186	-1,894
HLA-C;HLA-B	-0,621	-0,714
HLA-E	1,268	-2,038
HMG20A	0,907	-2,484
HMOX2	1,422	-1,910
HNRNPUL2;HNRNPUL2-BSCL2	1,136	-1,796
HOMER1	0,669	-2,476
HOXB6	-0,371	-2,262
HRSP12	1,277	-1,542
HSPA4	1,025	-1,939
HYI	1,743	-1,538
IFT22	0,700	-1,389
IFT57	0,757	-1,842
IKBKG	-0,085	-1,924
ILK	0,187	-2,227
IMPA1	0,691	-1,555
IMPAD1	2,185	-1,634
INPP4B	1,355	-2,209
ISG20L2	1,524	-1,762
ISYNA1	-0,174	-1,288
IWS1	0,376	-2,738
JMY	-0,370	-1,198
KBTBD11	0,199	-1,692
KCT2	0,795	-1,392
KCTD1	0,379	-1,552
KCTD14	0,775	-2,243
KIAA0586	0,355	-1,788
KIAA0930	1,351	-1,976
KIF1B	0,664	1,930
KIF3A	1,184	-1,290
KLHL11	0,290	-1,751
LDB1	1,228	-2,439
LEO1	0,472	-2,131
LMAN2L	0,887	-1,575
LPXN	0,908	-2,152
LRRC14	-0,142	-1,720
LZTS3	-1,080	-0,952
MAN2A1	1,515	-1,476

Gene symbol	Ctrl	56h
MAP1LC3A	0,352	-2,146
MAP2K4	2,171	-1,272
MAPK11	-0,057	-2,468
MAPK14	1,009	0,497
MAPK9	0,701	-2,360
MARCKSL1	0,896	-1,341
MARK2	0,860	-1,417
MATN3	0,831	-1,202
MCM8	1,583	-1,176
MED1	0,825	-1,752
MED17	0,755	-1,391
METTL2B;METTL2A	0,879	-2,192
METTL5	1,213	-2,059
MINPP1	0,752	-0,694
MITD1	1,507	-2,155
MLEC	0,516	0,051
MLLT6	0,421	-2,408
MMS22L	1,631	-1,463
MOB3A	0,715	-1,474
MRPL33	0,236	-1,356
MSI2	0,319	-1,831
MTA2	1,706	-1,319
MTERF3	1,288	-1,765
MTFR1L	0,708	-2,552
MTHFR	0,043	-1,439
MTM1	1,455	-1,609
MTMR2	1,097	-2,460
MTPN	0,190	-1,877
MVB12A	0,296	-1,934
MYL1;MYL3	0,682	-2,169
NAB1	1,538	-1,900
NADSYN1	1,044	-1,631
NAGA	0,967	-1,867
NAPB	0,296	-2,451
NDUFB8	1,340	-2,358
NFAT5	0,064	-0,931
NFKB1	1,841	-0,385
NFKBIE	-0,238	-2,176
NFYB	1,273	-1,776
NFYC	0,978	-2,048
NGEF	1,336	-1,816
NIPSNAP1	0,920	-0,821
NLRC5	-0,089	-2,501
NMD3	0,931	-1,995
NME2	1,131	-1,421

Gene symbol	Ctrl	56h	Gene symbol	Ctrl	56h
NMI	0,432	-1,094	PMM1	0,465	-1,732
NMT1	0,620	-2,125	POGK	-0,587	-1,861
NOP10	1,035	-2,327	POLG2	-0,626	-2,173
NOV	-0,070	-1,423	POLR3K	0,588	-2,699
NR2C2AP	0,990	-1,684	PPAT	0,291	-0,983
NSL1	0,282	-1,692	PPP1R12C	-0,019	-1,133
NSMAF	1,101	-2,230	PPP1R3B	0,529	-1,915
NSUN2	1,742	-1,666	PPP2R1B	1,494	-1,453
NUDT14	0,454	-2,330	PPP2R5C	0,759	-1,000
NUDT5	1,233	-1,997	PPP3R1	0,614	-1,877
NXT1;NXT2	0,856	-1,269	PPP5C	1,036	-1,847
OARD1	1,675	-2,133	PRAME	0,890	-1,388
OCIAD1	1,462	-1,468	PRKCH;PRKCE	1,035	-1,948
OGFR	1,479	-1,200	PRNP	0,778	-2,597
OIP5	1,101	-2,362	PRPF31	0,965	-1,962
OLA1	0,423	-1,393	PRPF38B	1,326	-1,585
OPA3	0,546	-1,673	PRPS1	1,847	-1,651
OPLAH	-1,146	-1,992	PRPSAP2	0,305	-2,154
OSCP1	0,543	-2,381	PRR14	0,566	-1,075
PALM3	1,313	-1,934	PRR14L	1,218	-1,998
PAN2	0,115	-1,902	PRRC2B	0,102	-2,002
PARP2	0,907	-1,866	PSMA1	0,594	-1,395
PAXBP1	2,150	-1,731	PSMC4	0,372	-1,438
PCBD1	0,067	-2,184	PSME3	1,489	-2,329
PCBP2	-0,754	-1,851	PTMA	0,171	-2,268
PCCA	1,247	-1,705	PTPN2	1,409	-2,442
PCDHGA4	0,340	-1,634	PTRH1	1,458	-1,977
PCTP	1,672	-1,259	PTRH2	0,217	-1,803
PDXP	1,330	-2,040	QDPR	0,734	-2,356
PFKL	0,685	-1,822	RAB43	0,829	-1,427
PGM2	0,667	-2,320	RABIF	-0,322	-1,588
PHC3	0,591	-2,223	RAC2	0,897	-1,895
PHF20L1	0,702	-1,597	RAC3	0,792	-1,863
PHF3	1,523	-1,778	RASSF2	0,777	-1,278
PIP4K2A	-0,008	-2,577	RBBP5	0,842	-2,128
PIP4K2C	0,537	-1,998	RBM27	1,246	-2,105
PIPSL	1,310	-1,959	RBMX2	0,986	-1,590
PIR	0,392	-2,192	RC3H1	0,040	-0,475
PITPNA	1,319	-1,550	RCOR3	-0,284	-2,423
PLAUR	0,558	-1,837	REXO2	1,097	-2,042
PLCB3	1,805	-1,512	RFXANK	0,248	-1,092
PLEKHA3	1,154	-1,662	RHOA	0,926	-1,682
PLEKHB2	0,661	-1,425	RILPL2	0,930	-2,243
PLEKHF2	0,754	-0,970	RIPK1	1,067	-2,118
PLEKHG5	1,269	-1,379	RNF138	1,298	-1,795

Gene symbol	Ctrl	56h	Gene symbol	Ctrl	56h
RNF20	0,969	-1,819	SPTY2D1	1,141	-2,286
RNF7	0,165	-2,600	SQSTM1	0,288	-1,725
RNMT	0,789	-2,455	SRPX	1,840	-0,997
RNPS1	0,547	-2,034	SS18	1,122	-2,039
RPE	-0,014	-1,571	SSH1	0,453	-2,207
RPRD1A	-0,104	-2,376	STAU2	1,194	-1,884
RPS27L;RPS27	1,229	-1,802	STK3	1,757	-1,779
RPS29	0,444	-2,671	STK38L	0,219	-2,464
RSRC2	0,588	-2,644	STXBP1	1,307	-2,124
RUFY2	0,938	-2,367	STYX	1,115	-1,670
RUVBL1	1,190	-1,343	SULT1C2	0,905	-2,216
SAMD4A	-0,052	-0,749	SURF4	1,153	-2,451
SAMD4B	1,150	-2,361	SUZ12	1,688	-1,179
SAP130	0,067	-2,232	SVIL	0,162	-1,642
SCFD1	0,830	-1,969	SYNE1	1,520	-1,359
SCRN2	0,939	-1,174	TADA1	0,214	-1,899
SDE2	0,143	-2,096	TAF9B	1,448	-1,783
SDF2	0,559	-2,542	TAGLN2	1,078	-1,238
SDSL	0,031	-1,406	TAOK1	1,171	-1,660
SEC61B	0,752	-2,304	TBC1D10B	1,443	-1,144
SECISBP2	0,329	-1,595	TBC1D25	1,076	-1,808
SECISBP2L	0,839	-2,251	TBC1D5	0,859	-2,105
SEH1L	1,247	-2,066	TBCEL	0,903	-1,448
SEPHS1	-0,681	-1,448	TBX15	2,744	-0,913
SEPHS2	0,678	-2,290	TCEB1	0,990	-1,815
SEPN1	1,261	-1,549	TDP2	2,292	-1,203
SERPINB5	0,874	-1,621	TEN1	0,717	-2,298
SET	0,556	-1,837	TGFBRAP1	-0,115	-1,971
SF1	-0,131	-0,288	TH1L;NELFCD	0,897	-1,752
SFMBT1	1,222	-2,058	THAP11	1,046	-1,954
SH3KBP1	0,265	-2,451	THG1L	0,901	-2,418
SHB	1,112	-1,490	THOC5	0,815	-1,148
SIMC1	1,498	-1,860	THOC7	0,384	-2,262
SIPA1L1	0,789	-2,112	THUMPD2	-0,044	-1,121
SIPA1L2	0,869	-2,061	TIMM10B	0,621	-2,681
SIX4	0,647	-2,703	TMED10	0,875	-1,165
SLC27A3	1,297	-1,332	TNIK	0,919	-2,281
SLC38A10	1,711	-1,217	TNK2	0,922	-1,608
SLC7A1	0,142	-2,366	TNRC6B	0,516	-1,905
SMARCA4	1,031	-1,729	TOM1L2	0,659	-1,483
SMYD3	1,342	-1,481	TOMM22	1,010	-1,176
SNRNP27	0,800	-2,669	TOR3A	0,780	-1,431
SON	1,202	-2,455	TPMT	1,175	-1,994
SOX13	0,721	-2,711	TRAPPC13	1,423	-1,984
SP140L	0,248	-2,136	TRAPPC2B;TRAPPC2	0,921	-2,210

Gene symbol	Ctrl	56h	Gene symbol	Ctrl	56h
TRDMT1	1,776	-1,472	WASF2	0,442	-1,719
TRIP4	-0,273	-2,121	WBSCR22	1,108	-2,367
TRMT11	-0,019	-2,506	WDFY2	0,275	-1,435
TRMT44	1,046	-1,888	WDR37	0,465	-2,114
TSEN2	0,250	-2,468	WDR55	1,389	-2,302
TSTA3	0,535	-1,318	WDR77	0,385	-0,952
TTC26	1,890	-1,402	WEE1	1,397	-1,269
TTC27	1,583	-1,383	WHAMM	0,066	-2,677
TUBB8	-0,309	-1,869	WHSC2;NELFA	0,752	-1,296
TUBG2;TUBG1	0,890	-1,750	WIZ	0,586	-1,546
TXNRD3	0,145	-1,098	WRN	1,186	-2,378
UBA5	0,527	-2,719	WTIP	0,713	-2,026
UBE2I	0,419	-1,716	YBX3	1,071	-2,458
UBE2Z	1,121	-2,353	YTHDC1	1,233	-1,954
UBP1	1,076	-2,178	ZC3H7B	0,990	-1,996
UBQLN1	0,707	-1,820	ZDHHC7	0,628	-2,320
UBQLN4	1,332	-1,718	ZFP36L2	0,916	-1,428
UBXN1	0,310	-2,356	ZFYVE19	0,150	-1,772
UCC1;EPDR1	0,154	-1,108	ZFYVE21	0,441	-2,581
UCKL1	0,472	-1,804	ZFYVE9	0,913	-2,026
UPF3A	1,064	-2,400	ZNF131	0,841	-1,858
UPRT	0,968	-2,108	ZNF274	1,223	-1,700
USB1	1,342	-1,969	ZNF408	1,291	-2,015
USP16	0,528	-2,201	ZNF616;ZNF480	2,312	-0,891
USP47	1,135	-2,094	ZNF618	0,272	-2,139
VPS36	1,437	-2,158	ZNF622	0,566	-1,787
VTA1	1,022	-2,194	ZNF696	1,265	-1,449

Supplement Table 2: Yellow cluster of proteome analysis (upregulated at 56 h, log2 scale).

Gene symbol	Ctrl	56h
AARS	0,571	2,421
AATF	1,056	2,026
ABCC3	0,898	0,457
ACBD6	0,320	1,859
ACLY	0,306	2,349
ACTG1;ACTB;ACTA1	-1,773	1,490
ACTL6A	1,022	1,895
ACTR10	-0,210	2,082
ACTR3	0,393	1,996
ADA	-0,246	1,825
ADAM10	-0,070	1,720
ADAT1	0,473	1,705
ADAT3	-0,118	1,044
ADI1	0,625	1,106
ADNP	0,772	2,037
AES	0,191	0,389
AFF1	-0,726	2,486
AGL	-0,720	1,723
AHCY	0,696	1,827
AHCYL1	-0,061	1,784
AK2	-0,289	1,311
AKAP9	1,286	0,918
AKR1B1	0,288	1,186
ALDH1A2	-0,689	1,215
ALDH3A1	-0,542	1,833
ALPPL2;ALPP	-0,129	0,416
AMZ2	0,034	2,305
ANKZF1	-0,343	2,263
ANXA1	-0,625	1,625
APLF	-1,209	0,874
APOC3	-0,780	1,511
ARFGAP2	1,459	0,992
ARFGAP2	1,459	0,992
ARHGAP31	1,221	1,410
ARHGEF12	0,742	1,165
ARHGEF26	1,069	1,841
ARID4A	-0,022	0,791
ARL2	-0,969	2,519
ARL8A;ARL8B	-0,385	2,558
ARPC1B	0,757	0,706

Gene symbol	Ctrl	56h
ASH2L	1,580	1,023
ASPSCR1	0,191	1,353
ASS1	-0,252	1,958
ATAT1	0,137	1,141
ATF2	-1,198	2,062
ATF7IP	0,867	0,514
ATL2	-0,628	2,188
ATXN3	0,384	2,445
AVL9	1,144	1,337
BAG6	0,666	2,154
BBS1	-0,601	1,504
BLOC1S3	-0,733	2,254
BLOC1S4	-0,579	2,071
BMP1	-0,242	-0,496
BRAF	0,269	0,427
BRD1	0,615	-0,506
BRD2	1,137	0,849
BRE	-0,082	1,974
C10orf35	1,134	1,989
C12orf43	1,122	1,447
C15orf52	1,634	0,354
C1orf122	-0,088	1,946
C5orf45	0,191	1,545
C6orf203	0,202	1,405
CAAP1	0,595	2,365
CALB1	-0,528	1,665
CALD1	-1,690	0,333
CAPRIN1	0,576	2,048
CAPRIN2	-0,096	1,712
CAPZA2	-0,076	2,179
CARKD;FLJ10769	0,783	-0,121
CARS	-0,069	1,557
CASK	-0,666	1,623
CASKIN1	-1,135	1,285
CASP2	0,130	2,339
CASP6	-0,548	2,652
CBX1;CBX3	-0,579	2,699
CBX5	-0,096	2,095
CBX6	-0,568	1,331
CC2D1A	-0,330	1,905
CCAR2	0,927	2,063
CCBL1	0,391	2,091
CCDC47	0,169	2,303
CCDC6	-1,431	1,464

Gene symbol	Ctrl	56h
CCDC9	-0,474	2,595
CCT2	0,570	1,503
CCT8	-0,037	1,923
CD46	-0,032	-0,076
CD70	-0,592	0,582
CDC37	-0,235	2,638
CDH13	0,763	1,387
CDH6	-0,880	1,392
CDK12	0,012	2,130
CDK13	1,410	1,528
CDK20	-0,409	1,788
CEP104	0,911	2,285
CFDP1	-0,920	1,917
CHAC2	0,574	2,130
CHCHD7	-1,217	1,031
CHEK2	-0,021	2,635
CHFR	1,988	1,234
CHM	0,479	2,375
CHMP2B	-1,496	1,424
CHN1	-1,476	2,028
CHRAC1	-0,041	1,120
CHURC1-FNTB;FNTB	-1,084	2,394
CINP	0,560	1,706
CKB	-0,426	2,336
CLEC16A	-0,085	2,518
CLIC4	-1,506	2,069
CLK3	-0,486	1,687
CLOCK	-0,742	1,563
CMC4	-0,303	2,699
CNIH4	1,269	2,220
CNOT11	0,917	2,052
CNOT2	1,441	1,231
CNRIP1	-1,042	2,530
COL4A3BP	0,721	2,162
COL5A2	-0,637	1,921
COMMD1	-0,003	2,053
COMMD3-BMI1;BMI1;PCGF2	0,658	2,237
COQ7	0,745	1,985
CPOX	0,638	1,287
CPQ	-0,997	1,979
CRTC1	-1,051	1,283
CSF1	-1,352	0,976

Gene symbol	Ctrl	56h
CTNNBL1	0,866	2,212
CTSB	-1,124	2,130
CTSL	0,930	2,101
CXCL8	-0,086	1,418
CXXC5	-0,365	1,926
DARS2	0,911	1,720
DAZAP1	-0,072	2,335
DBNL	-0,975	2,342
DCAF15	0,670	2,523
DCLK1	-0,038	1,556
DCP2	0,517	2,041
DCTN6	-0,218	2,572
DDX1	0,613	2,087
DDX39A	0,335	2,379
DDX39B;DDX39A;hCG_2005638	-0,106	2,330
DDX3X;DDX3Y	0,863	1,532
DEK	1,146	1,464
DEPDC7	1,286	0,633
DES	-0,076	2,594
DHX36	0,654	1,867
DLST	1,152	1,538
DMAP1	1,139	2,245
DNAJC11	0,570	1,944
DNAJC7	0,936	1,786
DNTTIP2	0,871	1,486
DPH5	-0,254	2,626
DSC1	0,820	1,862
DSCC1	1,034	2,210
DSG1	0,242	2,558
DST	-0,566	2,047
DTNB;DTNA	-0,198	0,780
DYNC2LI1	-0,135	1,680
E2F4	0,829	2,071
E2F8	0,702	0,885
ECHS1	0,320	2,389
EHBP1	-0,116	2,692
EIF2A	-0,289	1,559
EIF2S1	0,668	2,430
EIF3B	0,672	2,189
ELOF1	-0,391	2,185
EMD	0,760	1,643
EML1	0,447	0,627

Gene symbol	Ctrl	56h
EMSY;C11orf 30	1,564	1,685
ENAH	-0,082	1,834
ENO3	-1,121	2,282
EP400	0,473	1,830
EPS15L1	-0,124	1,251
ERBB2	0,148	0,989
ERCC6	-1,057	1,191
ERF	0,272	0,452
EVI5L	0,465	0,461
EXOC6	0,520	1,338
FABP3	0,569	0,792
FAM136A	0,195	2,337
FAM168A	0,350	1,584
FAM175B	-0,213	2,538
FAM188B	-0,333	1,319
FAM83G	1,329	2,005
FAM96A	-0,255	2,209
FAM98B	0,149	1,861
FBL	1,262	1,529
FBRSL1	-1,333	1,210
FBXO3	-0,344	2,347
FH	0,787	2,050
FKBP11	1,410	1,188
FKBP1A;FKB P12-Exip2	-0,686	1,474
FLOT2	0,738	1,448
FOXK2	0,131	1,187
FTO	0,422	2,391
FUS	-0,041	1,464
FXR2	1,135	1,592
FZD6	-1,353	0,265
GAB1	-0,167	2,131
GALNS	0,470	0,960
GALNT1	0,218	1,004
GAN	-0,472	0,654
GATSL3	0,463	0,497
GBE1	-0,347	1,134
GCAT	0,734	2,076
GCC1	1,683	1,587
GCDH	0,276	2,495
GDF15	-0,788	1,404
GDI1	-0,180	1,841
GFPT2	0,923	2,140
GLB1	-0,546	1,445

Gene symbol	Ctrl	56h
GLDC	0,231	1,591
GLIPR2	0,044	1,995
GLRX	0,588	0,836
GNAI2;GNAT 1;GNAI1;GNA T2;GNAT3	-0,148	2,183
GNG11;GNG T1;GNGT2	1,197	0,411
GORAB	0,455	1,835
GPATCH1	0,313	1,375
GPI	0,087	2,120
GPN1	0,253	2,067
GPR108	-1,024	1,967
GPS2	0,070	2,673
GPX1	-0,688	2,299
GRN	-1,171	1,013
GRSF1	0,860	1,385
GSE1	-0,424	1,766
HAPLN3	-0,921	1,669
HAUS7	1,132	0,818
HAX1	0,368	1,240
HBA1	0,262	1,856
HBA2	0,028	1,933
HBG2;HBG1	-1,741	1,073
HBZ	-0,720	0,538
HCLS1	-0,932	1,829
HDDC2	-0,417	0,645
HDDC3	-0,792	1,789
HEBP1	-0,271	1,319
HIBADH	0,133	2,506
HIP1	-0,079	2,425
HIST1H1E;HI ST1H1D	1,012	1,148
HMGB2	-0,881	2,379
HMGN5	0,343	0,201
HMGXB3	-0,569	1,858
HN1	-0,395	2,259
HNF1A;HNF1 B;HMBOX1	0,430	2,418
HOXB4	-1,236	0,900
HP1BP3	-0,383	2,298
HPX	0,000	1,475
HR	0,002	2,470
HS1BP3	0,472	1,255
HSBP1	1,106	1,700
HSD17B8	-0,414	1,640

Gene symbol	Ctrl	56h
HSPA1A	-0,839	2,078
HSPA2	-0,859	2,271
HSPA4L	0,045	1,564
HSPBAP1	0,798	0,994
IAH1	-0,439	2,622
ICAM1	-1,009	1,545
IDH3A	0,790	1,901
IDI1	1,880	0,905
IFT52	-0,074	2,035
IL18	0,010	1,466
INVS	0,725	1,730
IQSEC2	0,013	2,069
IRF5	-0,170	1,609
IRS1	0,155	2,021
ITFG2	1,738	1,822
ITFG3	-0,490	1,191
ITGB4	1,264	1,042
ITGB8	0,500	1,028
ITPKA	-1,254	1,589
ITPKB	-0,268	1,554
KANK1	0,690	1,814
KCTD17	-0,081	2,554
KDM8	-0,709	1,326
KHNYN	-0,228	1,808
KIAA1161	0,100	1,905
KIAA1522	-0,753	1,771
KIAA1598	-1,031	1,516
KIDINS220	0,057	0,869
KIT	-0,303	0,178
KLC4	-1,229	1,186
L3HYPDH	0,079	1,676
L3MBTL3	0,305	1,677
LANCL2	0,135	2,325
LASP1	-0,798	1,628
LCORL	0,252	2,300
LCP1	-1,329	0,651
LDHB	-0,056	1,792
LDLR	0,420	0,565
LENG1	-0,290	2,388
LMAN1	-0,164	2,224
LMNB2	0,313	1,852
LPP	-1,443	1,350
LRRN1	-1,232	0,516
LSM12	1,603	1,157

Gene symbol	Ctrl	56h
LSM2	-0,306	2,699
MAK16	0,833	1,398
MAL2	1,332	1,283
MAN1A2	0,588	1,656
MAPRE1	0,162	2,234
MASP1	-0,293	2,096
MCTS1	-0,056	2,463
MED10	0,132	1,610
MED20	0,460	2,519
MED25	0,371	1,777
METRNL	0,953	0,987
MEX3D	-0,129	1,207
MGMT	-0,099	1,338
MIF4GD	-0,218	2,544
MISP	1,001	0,722
MKNK1;DKFZp686E14208	0,359	1,554
MMGT1	-0,489	1,667
MMP21	-0,500	1,740
MOB2	0,604	1,808
MPG	0,749	0,803
MPHOSPH8	-0,019	1,683
MPRIP	1,495	0,106
MPST	1,647	1,253
MRE11A	0,614	1,575
MRPS25	0,840	2,099
MRTO4	0,098	2,248
MTAP	1,402	1,074
MTDH	0,571	2,156
MVD	-0,589	2,358
MYDGF	-0,290	1,792
MYH14	0,480	1,700
MYH9	-0,991	1,893
MYL12A;MYL12B	-0,206	2,407
MYO1D	0,415	1,937
NAA10;NAA11	-0,152	2,451
NACA	-0,923	0,879
NAMPT	1,384	1,662
NAP1L4	-0,275	2,435
NAPG	-0,287	2,631
NCAPH	1,483	1,291
NCDN	0,508	1,712
NCK1	0,374	2,031

Gene symbol	Ctrl	56h
NCKAP5L	-1,032	1,932
NCOA1	0,307	2,477
NDUFB3	1,070	0,595
NEO1	0,491	1,259
NFKB2	0,187	2,183
NME1-NME2;NME2;NME1;NME2P1	-0,200	1,478
NOL3	1,014	1,219
NOVA1	-1,066	1,741
NPNT	-0,327	1,302
NRF1	0,534	2,021
NUDT12	0,518	0,380
NUDT18	-0,468	1,462
NUDT9	-0,926	2,042
NUMB	0,534	1,197
NUMBL	0,430	1,956
NUP43	0,013	2,447
OGDH	0,685	1,952
OGDHL	1,061	1,375
P2RX4	0,727	2,191
PANK3	0,674	-0,240
PAPOLA	0,661	1,288
PARD6B	-0,859	2,663
PCDHGA10	-0,983	2,028
PCMT1	-0,355	1,744
PDAP1	0,580	2,280
PDCD10	0,780	2,245
PDCD6	-0,055	1,698
PDCD7	0,408	0,872
PDHB	0,443	2,173
PDIA6	-0,875	2,323
PDLIM1	-1,027	1,490
PDXK	-0,147	2,152
PDZD8	1,301	1,015
PDZK1;PDZK1P1	0,221	1,529
PEF1	0,780	1,618
PFKFB3	-0,491	1,876
PHF21A	-0,172	0,912
PHPT1	-1,456	1,631
PKD2	-0,246	1,512
PKM	0,138	1,293
PLCG1	0,034	1,449

Gene symbol	Ctrl	56h
PLEC	1,559	0,039
PLEKHM1	-0,373	1,861
PLXDC2	0,342	-0,227
PLXNA1	0,536	0,606
PMM2	0,019	1,522
PNPO	0,295	2,429
POLR1D	0,474	2,045
POLR2F	-0,163	2,321
POLR2H	0,297	2,326
POU2F1;POU2F3;POU2F2	-0,375	2,177
PPME1	0,544	2,051
PPP1CC	0,896	1,752
PPP3CA	-0,262	2,397
PPT1	-0,535	2,366
PRDX1	-0,598	1,301
PRDX6	0,149	1,530
PREP	0,203	1,927
PRKAR1A	0,539	1,580
PRKRA	-0,459	2,321
PRMT3	0,758	1,935
PROSER2;C10orf47	0,482	1,469
PROSER3	0,404	1,906
PRPF3	-0,386	1,869
PRR5-ARHGAP8;PRR5;LOC553158;ARHGAP8	0,904	2,058
PSMA5	-0,163	2,648
PSMC3	-0,330	2,551
PSMC5	1,116	1,496
PSMD10	-0,405	1,478
PSMD11	0,455	2,363
PSMD3	0,314	2,374
PSME1	-0,406	2,347
PSTPIP2	0,919	1,186
PTMS	-1,837	1,717
PTPN6	-0,893	2,308
PTRF	-0,664	2,277
PUS3	0,495	1,721
PVRL2	0,192	2,230
PYCRL	0,469	2,356
RAB14	1,175	1,716
RAB1B;RAB1C	-0,686	2,764

Gene symbol	Ctrl	56h
RAB28	1,095	2,052
RAB30	-0,365	2,099
RAB5B	-0,259	2,586
RABEP2	-0,262	2,094
RABEPK	0,130	1,922
RALGAPB	1,440	0,638
RASSF8	0,580	1,788
RBBP4;RBBP7	0,211	2,080
RBM5	0,050	1,740
REEP3	0,228	2,230
RGL3	-0,142	1,915
RMDN1	0,290	1,127
RMND5A	-0,542	2,443
RNF135	-0,075	2,251
RNF146	-0,375	2,051
RPL10A	-0,081	1,516
RPL14	-0,184	1,415
RPL32	0,252	2,423
RPL5	-0,157	1,169
RPS15	0,216	2,002
RPS17L;RPS17	-0,420	2,015
RRAS2	0,196	2,329
RRNAD1	0,639	0,788
RTCB	1,063	0,821
RTN1	-1,366	1,458
RUFY1	0,690	-1,218
SCAMP1	-0,072	2,538
SCAMP2	1,237	1,034
SCAPER	-0,529	1,721
SCP2	0,016	0,012
SCYL3	-1,495	1,908
SEMA3B	-0,208	1,080
SERPINB9	0,312	1,387
SETD1A	-1,252	1,032
SETX	0,666	2,414
SF3A1	1,126	1,612
SF3A2	-0,433	2,023
SH2D4A	-0,666	0,532
SHPK	-0,092	1,255
SIN3A	0,395	2,073
SLC12A2	-0,054	0,758
SLC16A3	0,852	1,635
SLC34A3;SLC	-0,483	2,187

Gene symbol	Ctrl	56h
34A1		
SLTM	-0,978	1,891
SLX4	1,331	0,754
SMCR8	0,924	1,148
SND1	-0,104	1,758
SNIP1	-0,607	2,304
SNRPE	-0,163	2,487
SNRPF	0,949	0,940
SNRPG;SNRPGP15	-0,543	2,365
SNRPN;SNRPB	0,313	2,138
SNTA1	0,583	1,158
SNX2	-0,121	2,432
SORBS2	-0,337	2,685
SORBS3	-0,128	2,191
SPATS2	0,090	2,200
SPICE1	0,567	0,593
SRP19	-0,682	2,400
SRRM1	-0,053	2,677
SRRM2	0,846	0,631
SSBP1	-1,153	0,328
SSH2	-1,243	1,576
ST13P5;ST13	-0,876	1,497
STAM	0,127	2,151
STAMBP	-0,285	2,018
STK38	0,408	2,228
STXBP4	0,403	1,978
SUB1	-0,516	2,467
SUMO1	-0,264	1,654
SUV420H1	0,330	2,082
TACSTD2	-1,104	0,944
TAP1	-0,054	0,983
TARS	0,495	2,110
TATDN2	0,768	0,871
TBC1D16	-1,283	1,420
TBC1D20	0,341	2,068
TBC1D23	0,128	2,550
TBC1D4	0,854	0,536
TECR	0,841	0,502
TET2	0,434	2,176
TFE3	-0,379	1,469
TICAM1	-1,322	1,921
TIMP2	0,153	1,917
TK2	1,295	1,463

Gene symbol	Ctrl	56h
TLDC1	0,593	1,714
TLN1	-0,560	1,892
TMEM106B	-0,727	1,288
TMEM55B	0,009	1,605
TMOD3	-1,261	2,450
TMUB1	1,385	0,071
TOB2	-0,526	1,178
TOE1	1,122	1,580
TOM1	-1,190	2,269
TOM1L1	0,391	2,476
TOR1A	-0,074	2,548
TPD52L2	-1,007	1,689
TPM1	-0,646	0,046
TRAF3IP1	0,130	2,668
TRAPPC2L	-0,467	2,228
TRIM2	-0,813	1,330
TRIM22	-0,751	0,892
TRIM37	0,680	1,358
TRPT1	0,606	0,728
TSC22D3	-1,109	1,867
TSC22D4	0,139	1,813
TSSK4	-0,339	2,216
TTC9	-0,288	2,447
TUBA1C	0,404	2,132
TUBB4B	0,094	2,606
TUBB6	-0,211	2,774
TULP4	0,128	1,911
TWF1	0,761	1,584
UBA3	1,410	1,009
UBE2H	0,800	1,574
UBE2K	0,410	1,905
UBE2M	0,596	1,836
UBE2V2	0,070	2,477
UBE4A	0,812	1,317
UBL5	-0,259	1,103
UBL7	-0,870	0,599
UBXN2B	-0,710	1,436
UCHL3	0,535	2,586
USP20	0,391	2,231
USP48	0,639	2,132
UTP11L	0,517	1,394
VBP1	0,126	1,316
VGLL4	-0,827	1,605
VPS54	1,299	1,533

Gene symbol	Ctrl	56h
VRK1	0,573	2,119
VWF	0,514	0,814
WARS	-0,522	2,004
WTAP	-0,858	2,437
WWC3	-0,129	0,987
WWOX	0,474	2,152
XPC	-0,405	2,469
XPNPEP1	0,450	1,607
XRCC6BP1	-0,703	2,111
YAP1	-0,228	2,015
YEATS2	-0,210	1,208
YWHAB	-0,029	2,494
ZBTB45	-0,686	1,252
ZDBF2	0,514	2,357
ZFAND1	1,018	1,980
ZHX2	0,141	1,867
ZNF574	1,240	-0,151
ZNF579	-0,170	1,690
ZNF703	-0,186	2,233
ZNRF2	-0,024	1,826

Supplement Table 3: Cluster genes of Wnt-signaling pathway enrichment at 56 h (log2 scale).

Gene symbol	Description	Ctrl	56 h
EP300	Histone acetyltransferase p300	1,169	-2,379
PLCB3	1-phosphatidylinositol 4,5-bisphosphate phosphodiesterase beta-3	1,805	-1,512
CSNK1A1	Casein kinase I isoform alpha	1,030	-2,068
CTNNBIP1	Beta-catenin-interacting protein 1	0,344	-2,751
MAPK9	Mitogen-activated protein kinase 9	0,701	-2,360
CHD8	Chromodomain-helicase-DNA-binding protein 8	0,549	-2,500
DVL3	Segment polarity protein dishevelled homolog DVL-3	1,324	-1,681
PPP2R1B	Serine/threonine-protein phosphatase 2A 65 kDa regulatory subunit A beta isoform	1,494	-1,453
CSNK2A2	Casein kinase II subunit alpha	1,081	-1,736
RAC2	Ras-related C3 botulinum toxin substrate 2	0,897	-1,895
RAC3	Ras-related C3 botulinum toxin substrate 3	0,792	-1,863
RHOA	Transforming protein RhoA	0,926	-1,682
RUVBL1	RuvB-like 1	1,190	-1,343
PPP3R1	Calcineurin subunit B type 1	0,614	-1,877
PPP2R5C	Serine/threonine-protein phosphatase 2A 56 kDa regulatory subunit gamma isoform	0,759	-1,000
NFAT5	Nuclear factor of activated T-cells 5	0,064	-0,931
CSNK1E	Casein kinase I isoform epsilon	-1,380	-0,968

Supplement Table 4: Proteome analysis of MRTF-associated genes after 56 h (log2 scale). #NV: not detected in proteome

Gene symbol	Description	Ctrl	56 h
MYOCD	Myocardin	#NV	#NV
CDH5	Cadherin 5	#NV	#NV
MYL9	Myosin Light Chain 9	#NV	#NV
MIR206	MicroRNA 206	#NV	#NV
EP300	E1A Binding Protein P300	1,17	-2,38
SMYD3	SET And MYND Domain Containing 3	1,34	-1,48
RHOA	Ras Homolog Family Member A	0,93	-1,68
MICAL2	Microtubule Associated Monooxygenase, Calponin And LIM Domain Containing 2	1,82	-0,67
ATE1	Arginyltransferase 1	0,11	-2,32
KPNA3	Karyopherin Subunit Alpha 3	1,83	-0,40
MKL1	Megakaryoblastic Leukemia (Translocation) 1	1,28	-0,72
BRMS1L	BRMS1, Breast Cancer Metastasis Suppressor 1	0,50	-0,88
DNMT1	DNA Methyltransferase 1	0,61	-0,40
MKL2	MKL1/Myocardin Like 2	1,18	0,41
LIMA1	LIM Domain And Actin Binding 1	-0,71	-1,33
KPNB1	Karyopherin Subunit Beta 1	1,44	1,09
C1QTNF6	C1q And Tumor Necrosis Factor Related Protein 6	0,65	0,41
STAT3	Signal Transducer And Activator Of Transcription 3	0,83	0,64
TRIM27	Tripartite Motif Containing 27	0,47	0,73
CYR61	Cysteine Rich Angiogenic Inducer 61	-0,58	0,00
SRF	Serum Response Factor	-0,09	0,54
PALLD	Palladin, Cytoskeletal Associated Protein	-0,68	0,28

Supplementary Figure 1: Full versions of the cropped Western blot in Fig. 6B

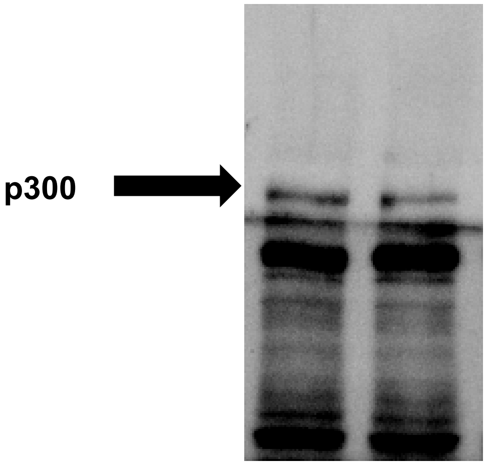

Complete blot for Figure 6B

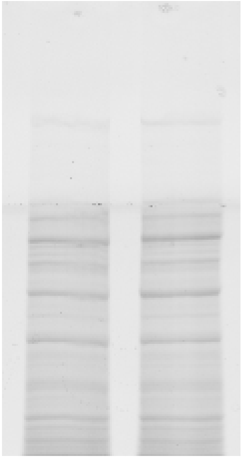

Loading control by stain-free gel for Figure 6B

Supplementary Figure 2: Dot plot of enriched pathways for up-regulated proteins

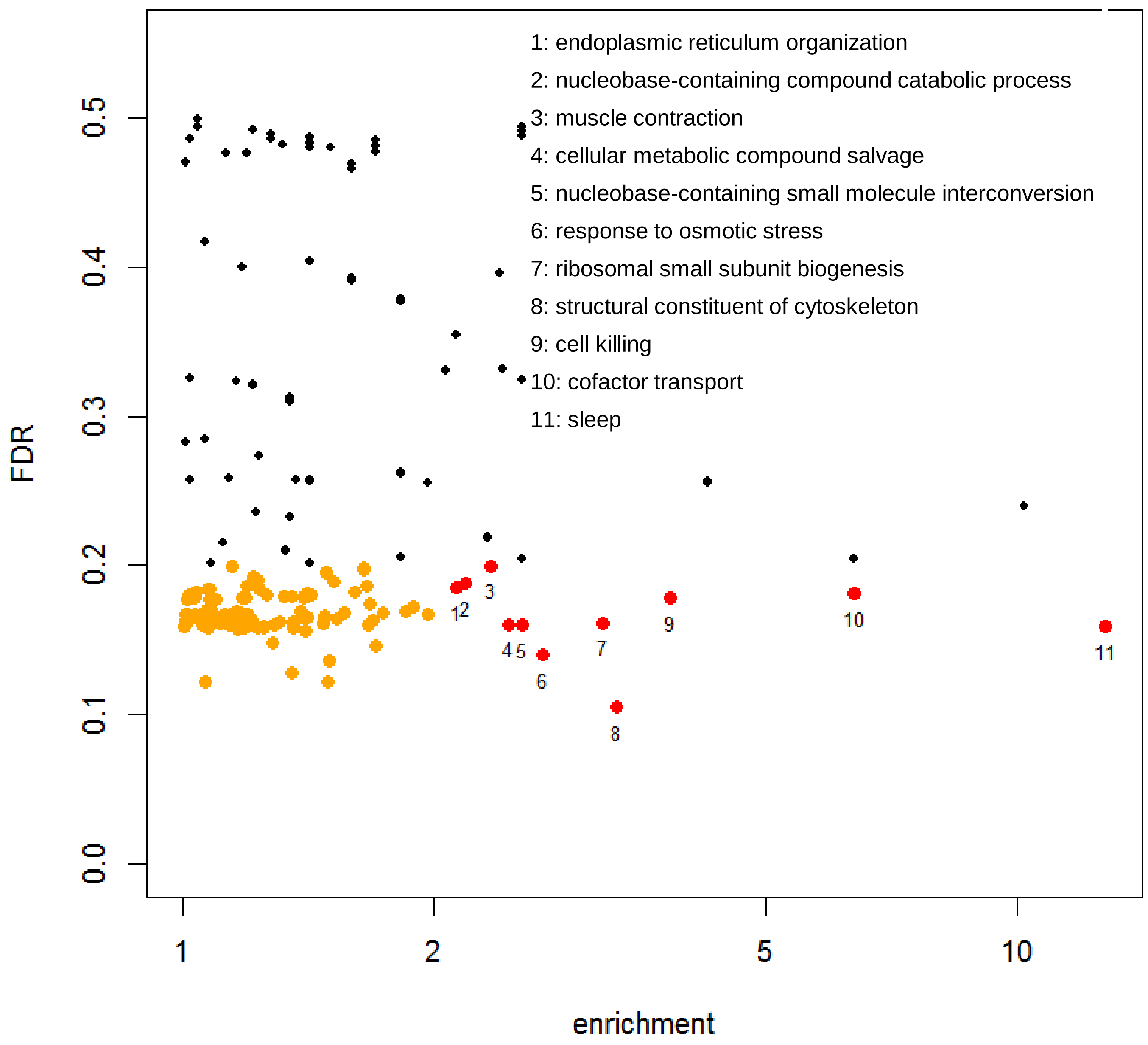

orange dots: FDR < 0.2 and enrichment value < 2.

red dots: FDR < 0.2 and enrichment value > 2.

Supplementary Figure 3: Viability, 2D and 3D migration of primary endothelial cells (HUVECs)

a: Viability

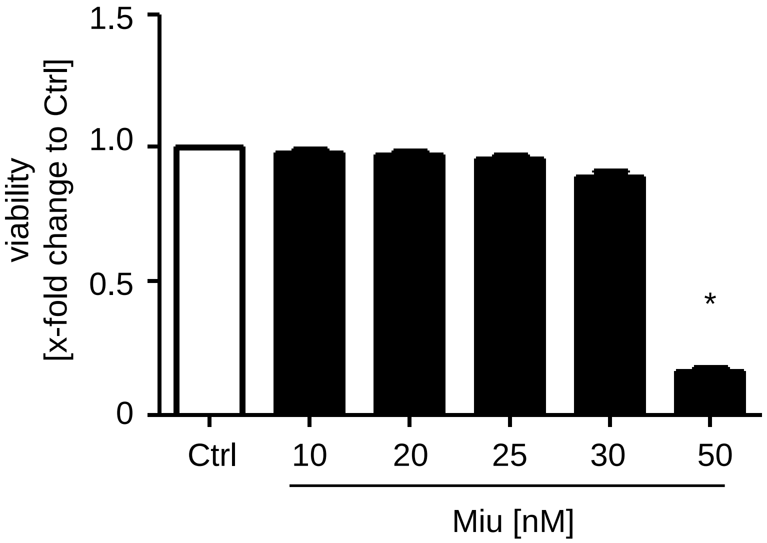

b: Scratch assay

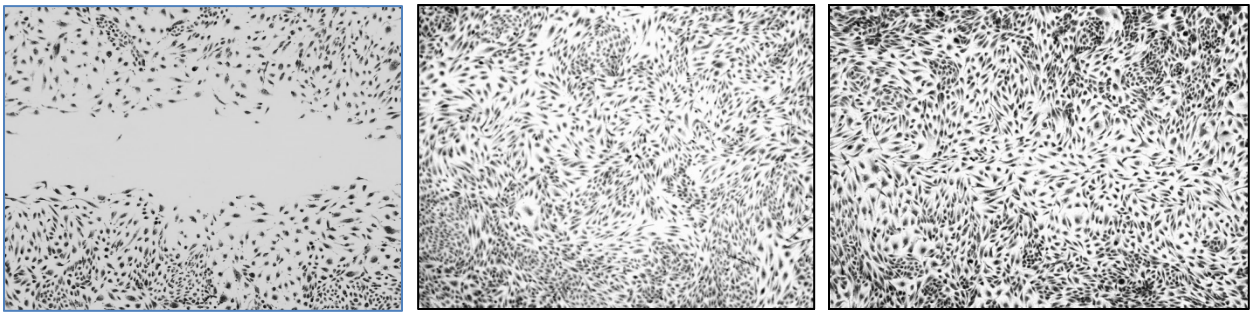

Negative control (minimal medium)

Positive control (ECGM)

Pretreatment with 20 nM Miu for 72 h

c: Boyden chamber

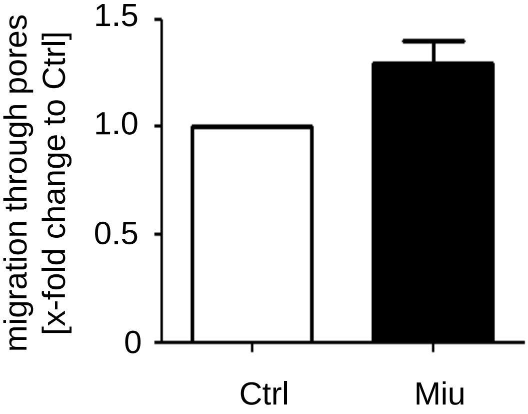

Supplement: Supplementary file 1 — Supplementary data [file 41598_2017_16759_MOESM1_ESM.pdf]
